# A General Approach to Direct Growth of Oriented Metal–Organic Framework Nanosheets on Reduced Graphene Oxides

**DOI:** 10.1002/advs.201901480

**Published:** 2020-01-03

**Authors:** Chao Liu, Xiaodan Huang, Jizi Liu, Jing Wang, Zibin Chen, Rui Luo, Chaohai Wang, Jiansheng Li, Lianjun Wang, Jingjing Wan, Chengzhong Yu

**Affiliations:** ^1^ School of Chemistry and Molecular Engineering East China Normal University Shanghai 200241 P. R. China; ^2^ Jiangsu Key Laboratory of Chemical Pollution Control and Resources Reuse School of Environmental and Biological Engineering Nanjing University of Science and Technology Nanjing 210094 P. R. China; ^3^ Australian Institute for Bioengineering and Nanotechnology The University of Queensland Brisbane QLD 4072 Australia; ^4^ Herbert Gleiter Institute of Nanoscience Nanjing University of Science and Technology Nanjing 210094 P. R. China; ^5^ Scitek Australia Pty Ltd Unit 1, 12 Chaplin Drive Lane Cove NSW 2066 Australia

**Keywords:** electrochemical application, graphene oxide, metal–organic frameworks, nanosheets

## Abstract

Ultrathin metal–organic framework nanosheets (UMOFNs) deposited on graphene are highly attractive, however direct growth of UMOFNs on graphene with controlled orientations remains challenging. Here, a low‐concentration‐assisted heterogeneous nucleation strategy is reported for the direct growth of UMOFNs on reduced graphene oxides (rGO) surface with controllable orientations. This general strategy can be applied to construct various UMOFNs on rGO, including Co‐ZIF, Ni‐ZIF, Co, Cu‐ZIF and Co, Fe‐ZIF. When UMOFNs are mostly attached perpendicularly on rGO, a 3D foam‐like hierarchical architecture (named UMOFNs@rGO‐F) is formed with an open pore structure and excellent conductivity, showing excellent performance as electrode materials for Li‐ion batteries and oxygen evolution. The contribution has provided a strategy for improving the electrochemical performance of MOFs in energy storage applications.

## Graphical abstract

Ultrathin metal–organic framework (MOF) nanosheets perpendicularly anchored on the surface of reduced graphene oxides with hierarchical architecture, open pore structure and excellent conductivity are generally synthesized through a low‐concentration‐assisted heterogeneous nucleation strategy, which show excellent electrochemical performances. This work provides a new strategy for improving the electrochemical performance of pristine MOF in energy storage applications.

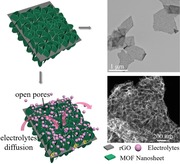

## 1 Introduction

Metal–organic frameworks (MOFs) are a new class of porous and crystalline materials with versatile functionalities.^1^, ^2^, ^3^ Their widespread applications have extended from gas adsorption,^4^ catalysis,^5^ drug delivery,^6^ sensor technology^7^ to templated synthesis of functional materials, such as porous carbons,^8^, ^9^ metal oxides,^10^, ^11^ and metal chalcogenides.^12^, ^13^ Pristine MOFs are also emerging as promising candidate materials in electrochemical applications.^14^, ^15^, ^16^, ^17^ However, several concerns including low electrical conductivity (usually ≈10^−10^ S m^−1^), large particle size and small pore size hamper their electrochemical applications, because poor electron transfer and slow mass transport lead to unsatisfactory electrochemical performance.^18^, ^19^, ^20^ It is of great significance to address the above drawbacks for electrochemical applications of MOFs‐based materials with high performance.

Very recently, 2D ultrathin MOF nanosheets (UMOFNs) have attracted specific research interest due to their faster mass transport and more accessible active sites compared to the conventional 3D bulk MOFs.^21^, ^22^, ^23^, ^24^, ^25^, ^26^, ^27^, ^28^, ^29^, ^30^ However, UMOFNs still have limitations including low electrical conductivity and monotonous micropores, hindering their applications in electrochemical fields.^31^, ^32^ Hybrid graphene‐based heterostructures, which utilize graphene as the substrate for growing other types of 2D nanosheets, can significantly improve the electrical conductivity of the “guest” sheets and further create 3D structures with hierarchical porous structure.^33^, ^34^, ^35^, ^36^ However, UMOFNs on graphene with a hierarchical hybrid structure have not been reported to the best of our knowledge. A close example is the deposition of 3D granular MOFs (e.g., zeolitic imidazolate frameworks, ZIF‐8 and ZIF‐67) on covalently functionalized graphene oxides (GO) through a homogenous nucleation strategy.^37^, ^38^, ^39^, ^40^ Up to now, it remains challenging to grow UMOFNs on GO sheets with controllable structures and enhanced electrochemical performance.

**Figure 1:**
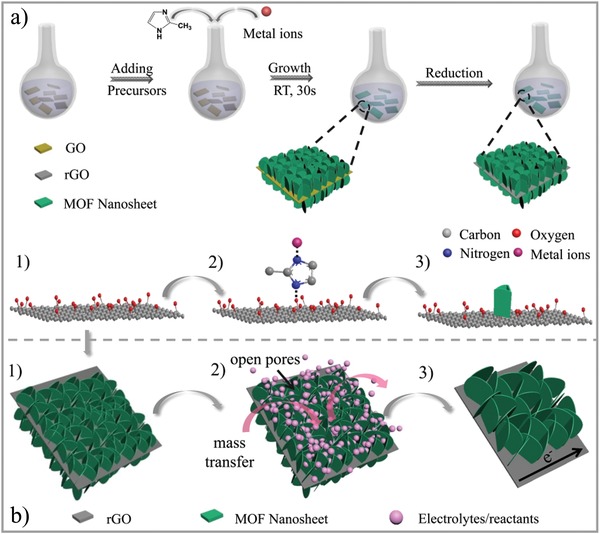
a) Schematic illustration of the preparation of UMOFNs@rGO‐F hybrids by a low‐concentration‐assisted heterogeneous nucleation pathway. b) Structural advantages of UMOFNs@rGO‐F hybrids for electrochemical applications.

**Figure 2:**
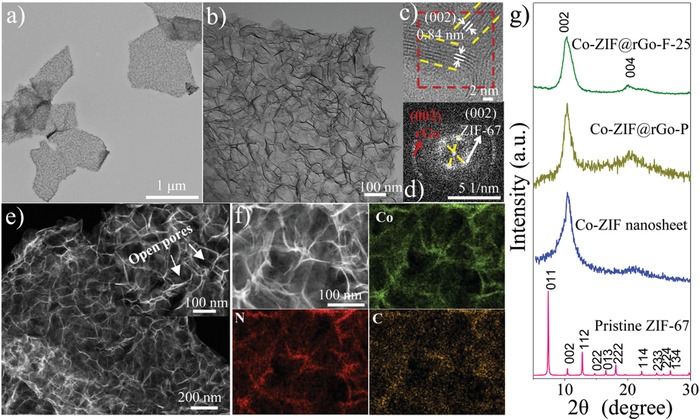
a,b) TEM images; c) HR‐TEM image; d) SAED patterns; e) HAADF‐STEM images; f) TEM image and element mapping of Co‐ZIF@rGO‐F‐25 hybrids. g) XRD patterns of pristine ZIF‐67, Co‐ZIF nanosheet, Co‐ZIF@rGO‐P, Co‐ZIF@rGO‐F‐25.

**Figure 3:**
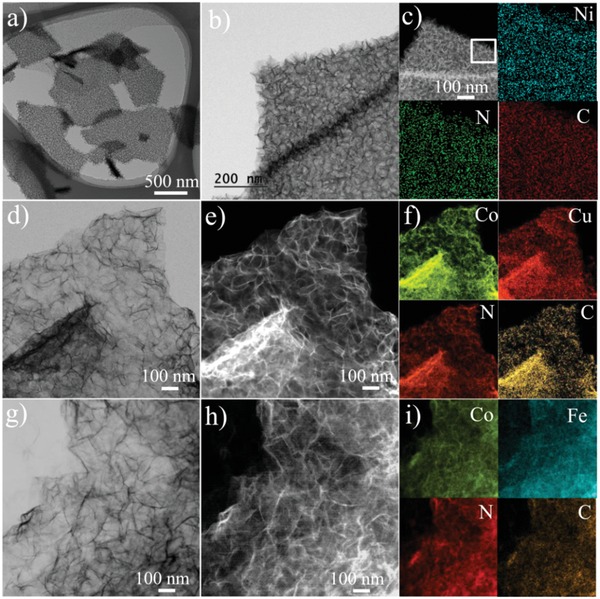
TEM, STEM, and element mapping images of a–c) Ni‐ZIF@rGO‐F, d–f) Co,Cu‐ZIF@rGO‐F, and g–i) Co, Fe‐ZIF@rGO‐F.

Herein, for the first time, we report the direct synthesis of ultrathin metal–organic framework nanosheets with perpendicular orientation deposited on reduced graphene oxides (UMOFNs@rGO‐F) by a low‐concentration‐assisted heterogeneous nucleation pathway (**Figure** **1**). The strategy can be generally applied to construct various UMOFNs on rGO, including Co‐ZIF, Ni‐ZIF, Co, Cu‐ZIF and Co, Fe‐ZIF. First, GO prepared by the modified Hummer's method were dispersed in methanol and water solution without further modification (Figure 1a‐1).^41^, ^42^ Then, the organic ligand, 2‐methylimidazole (2‐MeIM), was added at a low concentration and attached to the surface of GO due to the electrostatic attraction (Figure 1a‐2). Subsequently, the addition of metal ions (the same concentration as 2‐MeIM) resulted in 3D foam‐like UMOFNs deposited on the surface of GO by a heterogeneous nucleation pathway in a very short time of ≈30 s (Figure 1a‐3), forming UMOFNs@GO‐F. After partial reduction,^43^ hierarchically structured UMOFNs@rGO‐F was finally obtained with improved conductivity, abundant open pores and active sites (Figure 1b), showing excellent performance when used as electrode materials for Li‐ion batteries and oxygen evolution.

## 2 Results and Discussion

To demonstrate the structure characteristics and formation mechanism of UMOFNs@rGO‐F hybrids, Co‐ZIF@rGO‐F is chosen as a typical example. A series of Co‐ZIF@rGO‐F‐*x* samples were first fabricated using various precursor concentrations (*x* is the concentration of 2‐MeIM and Co^2+^, *x* = 5, 10, 15, 25, and 50 × 10^−3^ m). As shown in transmission electron microscopy (TEM) and atomic force microscopy (AFM) images (Figures S1 and S2, Supporting Information), by increasing *x* from 5 to 25 × 10^−3^ m, the density and sizes of Co‐ZIF nanosheets on rGO as well as the thickness of Co‐ZIF@rGO‐F‐*x* are gradually increased. The Co‐ZIF nanosheets are mostly grown on the surface of rGO, forming a 3D interconnected foam‐like network at 25 × 10^−3^ m. When the concentration is further increased to 50 × 10^−3^ m, aggregated nanosheets are found on the edge or even outside of rGO (Figure S1e, Supporting Information). These isolated nanosheets may retain the poor conductivity of blank UMOFNs due to the lack of connection with rGO, which are unfavorable for electrochemical applications.

The X‐ray diffraction (XRD) patterns indicate that the Co‐ZIF nanosheets in Co‐ZIF@rGO‐F‐*x* possess the same crystalline structure (Figure S3, Supporting Information). The background noise is more obvious in the XRD patterns of Co‐ZIF@rGO‐F‐5 to 15, indicating a lower content of Co‐ZIF in the composites. Inductive coupled plasma emission spectrometer was employed to quantify the contents of Co‐ZIF nanosheets in Co‐ZIF@rGO‐F‐*x*, which are calculated to be 16.2%, 39.5%, 56.1%, 82.3%, and 88.1% in Co‐ZIF@rGO‐F‐5, 10, 15, 25 and 50, respectively (Figure S1f, Supporting Information). Considering both the structure and content of active species, Co‐ZIF@rGO‐F‐25 is chosen as the optimized sample.

The structures of Co‐ZIF@rGO‐F‐25 were extensively investigated. Compared to the as‐synthesized GO with a nanosheet structure and smooth surface (Figure S4a, Supporting Information),^44^, ^45^ micron‐sized composite nanosheets with rough surfaces can be observed exclusively after growth of Co‐ZIF nanosheets (**Figure** **2**a and Figure S5, Supporting Information). TEM images at higher magnification show that a large number of 2D ultrathin nanosheets are uniformly anchored mostly perpendicularly on the surface of rGO (Figure 2b). The high‐angle annular dark‐field scanning TEM (STEM) images further reveal that these nanosheets are relatively vertically oriented on rGO and interconnected with each other, giving rise to a well‐developed 3D foam‐like hierarchical network with rich open pores (Figure 2e). The thicknesses of Co‐ZIF@rGO‐F‐25 and Co‐ZIF nanosheet are determined to be ≈25–35 and ≈6.2 nm, respectively (Figure S6, Supporting Information). In contrast, under the same reaction conditions of Co‐ZIF@rGO‐F‐25 but without GO, 2D Co‐ZIF nanosheets with sizes of ≈120 nm were obtained without a foam‐like morphology formed (Figure S7, Supporting Information). When the Co‐ZIF was removed by immersing Co‐ZIF@rGO‐F‐25 in 1 m HCl, rGO nanosheets with smooth surface were observed, similar to pristine GO (Figure S8, Supporting Information). These results further indicate that the 3D foam‐like structure is formed by the growth of Co‐ZIF nanosheets on rGO.

In order to confirm the crystal structure of Co‐ZIF nanosheets on Co‐ZIF@rGO‐F‐25, the XRD characterization was performed (Figure 2g). The XRD pattern of Co‐ZIF@rGO‐F‐25 shows only two diffractions corresponding to the (002) and (004) facets of ZIF‐67,^46^ suggesting the Co‐ZIF nanosheets have a preferred orientation. This observation is similar to previous reports that ultrathin MOF nanosheets only show an oriented plane compared to bulk MOF material in their XRD pattern.^28^, ^46^ For comparison, the XRD pattern of rGO shows a broad diffraction peak at ≈23°–24° (Figure S9, Supporting Information), in accordance with previous reports.^47^, ^48^ In addition, the XRD pattern of α‐Co(OH)_2_ (prepared as a control, see the Supporting Information) displays three major peaks at 9.6°, 19.2°, and 33.7°, which can be assigned to (003), (006), and (009) diffractions of a hydrotalcite‐like structure (JCPDS No.51‐0916).^49^, ^50^ These results indicate that the two diffractions in Co‐ZIF@rGO‐F‐25 should be assigned to Co‐ZIF, rather than rGO or α‐Co(OH)_2_.

In addition, a reported precipitation‐aggregation method,^28^ which may change the orientation of UMOFNs, was used to treat Co‐ZIF@rGO‐25‐F. The XRD pattern (Figure S10, Supporting Information) of collected sample is similar to that of Co‐ZIF@rGO‐25‐F prepared using the direct washing/drying method, suggesting the preferred orientation of Co‐ZIF nanosheets on rGO is hardly changed.

The high resolution TEM image (Figure 2c) shows clear fringes with interplanar distance of 0.84 nm, consistent with the *d*‐spacing value (0.85 nm) of (002) facets calculated from the XRD pattern of ZIF‐67, suggesting the Co‐ZIF nanosheets growth on GO substrate is predominately perpendicular to the [002] direction. The selected‐area electron diffraction (SAED) pattern gives two groups of decussate diffraction spots, corresponding to the (002) lattice planes from two decussate Co‐ZIF nanosheets. The outer diffraction ring with the indexed *d* value of ≈0.34 nm can be attributed to the (002) planes of rGO (Figure 2d). The results of HR‐TEM images and SAED pattern are consistent with the XRD observations, confirming the Co‐ZIF nanosheets have a preferred orientation with the (002) facets perpendicular to the GO substrate. In addition, the thickness of Co‐ZIF nanosheets is measured to be ≈5 nm, slightly smaller than the AFM results.

The elemental mapping results of Co, N, and C from a selected area of Co‐ZIF@rGO‐F‐25 show that the three elements are evenly distributed in the whole area especially where Co‐ZIF nanosheets are located (Figure 2f). N_2_ sorption isotherms and pore size distribution curves further demonstrate the hierarchical porous structure of Co‐ZIF@rGO‐F‐25 (Figure S11, Supporting Information). The UV–vis adsorption bands at 584 and 540 nm are characteristic of Co^2+^ in tetrahedral coordination (Figure S12a, Supporting Information), indicating the same coordination environment of Co^2+^ in both pristine ZIF‐67 and Co‐ZIF nanosheets of Co‐ZIF@rGO‐F‐25.46 Meanwhile, pristine ZIF‐67 and Co‐ZIF@rGO‐F‐25 display similar FT‐IR spectra (Figure S12b, Supporting Information). The bands at 1584 and 440 cm^−1^ correspond to the stretching mode of C=N and Co—N bonding. The Raman and XPS spectra also show that the Co‐ZIF in Co‐ZIF@rGO‐F‐25 possesses the same functional groups and chemical binding status as those in pristine ZIF‐67 (Figures S13 and S14, Supporting Information).^46^ Taken together, it is concluded that 3D foam‐like ultrathin Co‐ZIF nanosheets grown on rGO have been successfully synthesized. The structure and morphology of Co‐ZIF@rGO‐F are distinctly different from the granular MOF particles attached GO composites in previous reports.^37^, ^38^, ^39^, ^40^

To gain insight into the formation mechanism of Co‐ZIF@rGO‐F, systematic investigations have been carried out. By adding 2‐MeIM and Co^2+^ at a high concentration of 500 × 10^−3^ m into the GO solution, granular ZIF‐67 particles were sporadically anchored on the surface of GO (Figure S15, Supporting Information), indicating the use of low‐concentration precursors is essential for the heterogeneous nucleation and formation of nanosheets. When 2‐MeIM and Co^2+^ (25 × 10^−3^ m) were added into the solution before the addition of GO, Co‐ZIF nanosheets were formed through homogenous nucleation and then deposited onto GO, thus uniform and homogenous coating of Co‐ZIF nanosheets on GO cannot be formed (Figure S16, Supporting Information). Similar observations were found when adding Co^2+^ first and then 2‐MeIM (25 × 10^−3^ m) into the GO dispersion (Figure S17, Supporting Information), suggesting the interaction between GO and 2‐MeIM is stronger than that between GO and Co^2+^. The peaks assigned to 2‐MeIM at 3150 cm^−1^ (C—H bonds in methyl group) and 1118 cm^−1^ (C—N bond) were observed in the FT‐IR spectrum (Figure S18a, Supporting Information) of GO after adsorption of 2‐MeIM (GO/2‐MeIM), indicating the existence of 2‐MeIM. However, typical peaks (3200–2400 cm^−1^ for N—H···N hydrogen bond and 1843 cm^−1^ for N–H stretching vibration) related to the N—H bond in 2‐MeIM were not observed in GO/2‐MeIM (Figure S18b,c, Supporting Information), suggesting that the inter‐molecular hydrogen‐bonding of 2‐MeIM could be replaced by interaction between negatively charged epoxy/carboxylic groups of GO^51^ and positively charged 2‐MeIM.^52^ When rGO was used (Figure S4b, Supporting Information) with most surface functional groups eliminated (Figure S19, Supporting Information), Co‐ZIF nanosheets were generated in the solution by homogenous nucleation and attached parallelly on rGO (Figures S20 and S21, Supporting Information), forming a sample named Co‐ZIF@rGO‐P with a low Co‐ZIF content of 42.1%. In addition, the TEM images of freeze‐dried sample show the similar 3D foam‐like hierarchical structure (see details in Figure S22, Supporting Information), indicating the UMOFNs are grown onto GO substrates in the reaction solution.

The above results demonstrate that the use of GO and low concentrations (<50 × 10^−3^ m) of precursors with programmed addition (GO first, 2‐MeIM second and Co^2+^ at last) are essential for the super‐fast and heterogeneous nucleation of Co‐ZIF nanosheets uniformly anchored on GO surface with relatively vertical orientation. The orientation favors an interconnected 3D foam‐like network of Co‐ZIF@rGO‐F‐25 with a higher Co‐ZIF content of 82.3% than Co‐ZIF@rGO‐P with the flat orientation (42.1%), beneficial for applications where MOFs are the active components.

The thermal and chemical stability of Co‐ZIF@rGO‐F‐25 was further examined according to reported protocols.^53^ Thermal gravimetric analysis (TGA) profile (Figure S23, Supporting Information) shows a weight‐loss step of 8.6% (25–185 °C) associated with the loss of solvent molecules. The steep weight‐loss stage of 33.4% (185–260 °C) can be attributed to the decomposition of Co‐ZIF, in accordance with a previous report.^46^ The chemical stability of Co‐ZIF@rGO‐F‐25 was investigated by suspending samples in boiling methanol, water and aqueous sodium hydroxide solution. XRD patterns of samples after treatment show that Co‐ZIF@rGO‐F‐25 has an excellent stability in boiling methanol up to 7 days (Figure S24a, Supporting Information). However, Co‐ZIF was fully converted into CoOOH (JCPDS no. 07‐0169) and Co_3_O_4_ (JCPDS no. 43‐1003) in both boiling water and boiling 0.1 m NaOH/KOH after 1 day (Figure S24b–d, Supporting Information), implying poor hydrothermal stability.

To confirm the versatility of our strategy, three other types of UMOFNs were grown on the surface of rGO. **Figure** **3**a–c represents the typical TEM images and STEM image of Ni‐ZIF@rGO‐F, in which 3D foam‐like UMOFNs with vertical orientation are clearly observed. The element mapping result shows the uniform distribution of carbon, nitrogen, and nickel. The XRD pattern displays two broad peaks at 10.3° and 20.6° (Figure S25a, Supporting Information), consistent with the nanosheet morphology. The synthesis can be further extended to bimetal UMOFNs systems. Two kinds of UMOFNs were also vertically deposited on the surface of rGO, resulting in the formation of Co, Fe‐ZIF@rGO‐F and Co, Cu‐ZIF@rGO‐F (Figure 3d–I and Figure S25b,c, Supporting Information). In these two samples, the heterogeneous metal ions (e.g., Cu or Fe) can be either chemically trapped with N‐enriched defective structures or physically trapped in the microporous structures in the Co‐ZIF framework, similar to the bulk bimetal ZIF materials.^54^, ^55^ Therefore, the low‐concentration‐assisted heterogeneous nucleation strategy can be generally applied to synthesizing different types of UMOFNs@rGO‐F hybrids.

**Figure 4:**
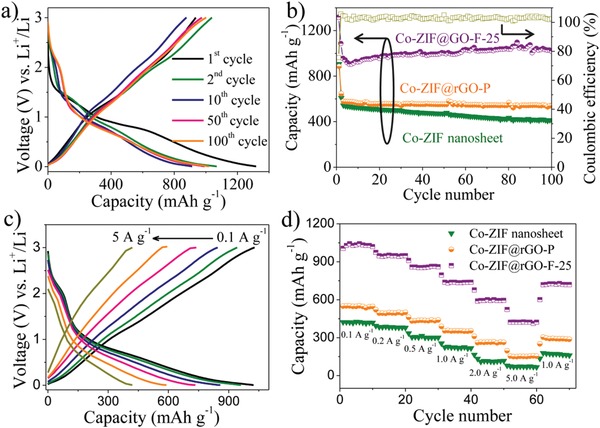
a) The representative galvanostatic charge‐discharge profiles of Co‐ZIF@rGO‐F‐25 at a current density of 0.1 A g^−1^; b) The cycling performances of Co‐ZIF nanosheet, Co‐ZIF@rGO‐P, Co‐ZIF@rGO‐F‐25 at a current density of 0.1 A g^−1^; c) Galvanostatic charge‐discharge profiles of Co‐ZIF@rGO‐F‐25 at various current densities; d) The rate performances of Co‐ZIF nanosheet, Co‐ZIF@rGO‐P, Co‐ZIF@rGO‐F‐25.

To demonstrate the application potential of UMOFNs@rGO‐F, Co‐ZIF@rGO‐F‐25 as a typical sample was first used as anode materials for Li‐ions batteries. Their performances were compared with Co‐ZIF@rGO‐P and pure Co‐ZIF nanosheets. The electrochemical performances were examined in coin‐cell batteries with Li foils as counter electrodes. The galvanostatic charge‐discharge profiles of Co‐ZIF@rGO‐F‐25 at 100 mA g^−1^ in the voltage window of 0.01–3.0 V are shown in **Figure** **4**a. At the first cycle, Co‐ZIF@rGO‐F‐25 demonstrates a high discharge and charge capacity of 1314.5 and 934.4 mAh g^−1^, respectively, with an initial coulombic efficiency of 71.1%. The excessive discharge capacity could be ascribed to the formation of solid electrolyte interphase (SEI) on the electrode surface.^45^, ^56^ From the second cycle onward, the coulombic efficiency has been significantly improved and reaches nearly 100% (Figure 4b), suggesting the highly reversible Li storage process. In addition, a stable cycling performance is observed in 100 cycles with a high reversible capacity of 1024.1 mAh g^−1^ at 100th cycle, which is 94.3% of the capacity at the second cycle (Figure 4b). When increasing the mass loading of active materials to 2.35 mg cm^−2^, Co‐ZIF@rGO‐F‐25 delivered the similar capacity of 1003.2 mAh g^−1^ after 100 cycles, indicating the excellent scaled performance (Figure S26, Supporting Information).

For comparison, pristine rGO electrodes were also tested with a poor capacity of only 127.5 mAh g^−1^ at 50th cycle, indicating that Co‐ZIF nanosheets in Co‐ZIF@rGO‐F‐25 are dominant active species (Figure S27, Supporting Information). Co‐ZIF@rGO‐P and pure Co‐ZIF materials also show inferior cycling performances. Their initial discharge capacities at 100 mA g^−1^ are 890.2 and 920.3 mAh g^−1^ (Figures S28 and S29, Supporting Information), respectively, lower than that of Co‐ZIF@rGO‐F‐25. After 100 cycles, Co‐ZIF@rGO‐P maintains a low reversible capacity of 542.5 mAh g^−1^ (83.1% of the capacity at the second cycle, (Figure S28, Supporting Information). The cycling stability of pure Co‐ZIF nanosheets is even inferior to that of Co‐ZIF@rGO‐P, showing only 408.2 mAh g^−1^ at 100th cycle with a poor retention of 67.5% (Figure S29, Supporting Information).

The rate performances were also investigated by cycling the battery at different current densities. As shown in Figure 4c, the reversible discharge capacities of Co‐ZIF@rGO‐F‐25 vary from 1022.8, 949.3, 864.1, 736.2, 592.6, and 418.4 mAh g^−1^ with the increasing current density from 0.1, 0.2, 0.5, 1, 2 to 5 A g^−1^, respectively. As the current density returns back to 1.0 A g^−1^, the capacity recovers to 718.4 mAh g^−1^ (Figure 4d) with an excellent retention of 97.4%. The rate performances of Co‐ZIF@rGO‐P and Co‐ZIF nanosheets electrodes were also tested. The reversible capacities of these two materials at different current densities are lower than the capacities of Co‐ZIF@rGO‐F‐25 (Figure 4d). At a high current rate of 5 A g^−1^, the capacities of Co‐ZIF@rGO‐P and Co‐ZIF nanosheets are 154.8 and 73.5 mAh g^−1^, respectively, which are only 21.8% and 10.3% of that for Co‐ZIF@rGO‐F‐25.

The electrochemical behaviors of these three samples were further investigated by electrochemical impedance spectroscopy (EIS). As shown in Figures S30 and S31 (Supporting Information), both the charge‐transfer resistance and Warburg impedance of Co‐ZIF@rGO‐F‐25 are lower than those of Co‐ZIF@rGO‐P and Co‐ZIF nanosheets, which can be ascribed to the direct growth of Co‐ZIF nanosheets on rGO substrates. The results also indicate that Co‐ZIF@rGO‐F‐25 with rich open pores and rGO as conductive substrate can provide abundant contact space for the access of electrolyte, accelerate charge transfer and promote Li‐ion diffusion, resulting in the significant enhancement of Li storage.

**Figure 5:**
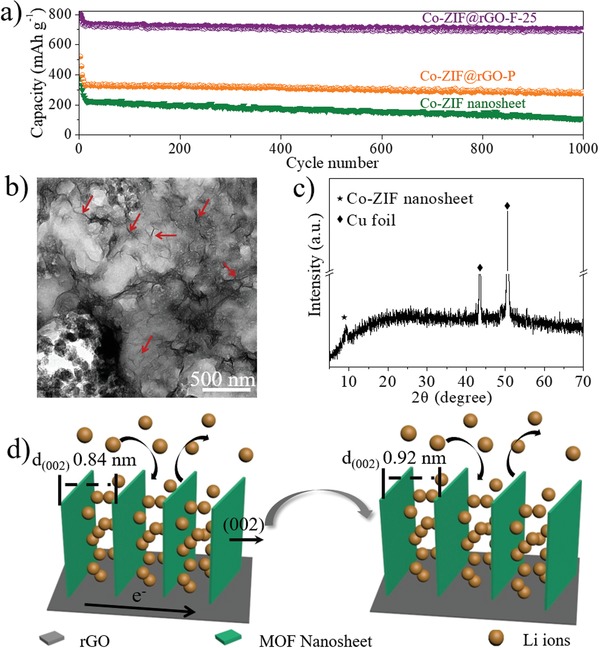
a) Long‐term cycling performance of Co‐ZIF nanosheet, Co‐ZIF@rGO‐P, Co‐ZIF@rGO‐F‐25 at a higher current density of 1 A g^−1^, b) TEM image, c) The XRD pattern of Co‐ZIF@rGO‐F‐25 based anode after cycling test; d) Schematic illustration of the interplanar distance of Co‐ZIF nanosheets in Co‐ZIF@rGO‐F‐25.

At a high current rate of 1 A g^−1^, Co‐ZIF@rGO‐F‐25 shows a stable capacity of 713.2 mAh g^−1^ after 1000 cycles (**Figure** **5**a), which is much higher than that of Co‐ZIF@rGO‐P (274.0 mAh g^−1^) and Co‐ZIF nanosheets (102.7 mAh g^−1^), further implying the excellent cycling performance and stability of Co‐ZIF@rGO‐F. The electrochemical performances of Co‐ZIF@rGO‐F‐25, including the reversible capacity, rate capability and cycling stability, are superior to Co‐ZIF@rGO‐P, Co‐ZIF nanosheets and most reported MOFs materials based anodes in previous reports (Table S1, Supporting Information).

The structure of Co‐ZIF@rGO‐F‐25 anode after cycling test was characterized in order to understand the relationship between the superior performance and nanostructure. In the TEM image (Figure 5b), the foam‐like ultrathin nanosheet network on rGO can be directly observed, indicating that the structure of Co‐ZIF@rGO‐F‐25 is well kept. As shown in Figure 5c, a typical peak corresponding to the (002) plane of Co‐ZIF nanosheet can still be observed, implying that the crystal structure of Co‐ZIF@rGO‐F‐25 is well remained during charge/discharge process. However, the diffraction peak is shifted from 10.4° to 9.5°, indicating that the interplanar distance of Co‐ZIF nanosheet in Co‐ZIF@rGO‐F‐25 anode after cycling test is increased (0.92 nm) compared to pristine Co‐ZIF@rGO‐F‐25 (0.84 nm). The similar expansions of (002) interplanar distance of Co‐ZIF nanosheets were also observed in the electrodes of Co‐ZIF@rGO‐P and Co‐ZIF nanosheets after cycling test (Figure S32, Supporting Information). Previous reports on MOF based LIB anodes have revealed that the nitrogen atoms in imidazole‐type ligands can interact with Li^+^ and provide insertion sites for Li storage.^56^, ^57^ Therefore, the enlargement of interplanar distance of Co‐ZIF nanosheet (Figure 5d) may be due to the insertion and de‐insertion of Li^+^ in the interplanar space.

**Figure 6:**
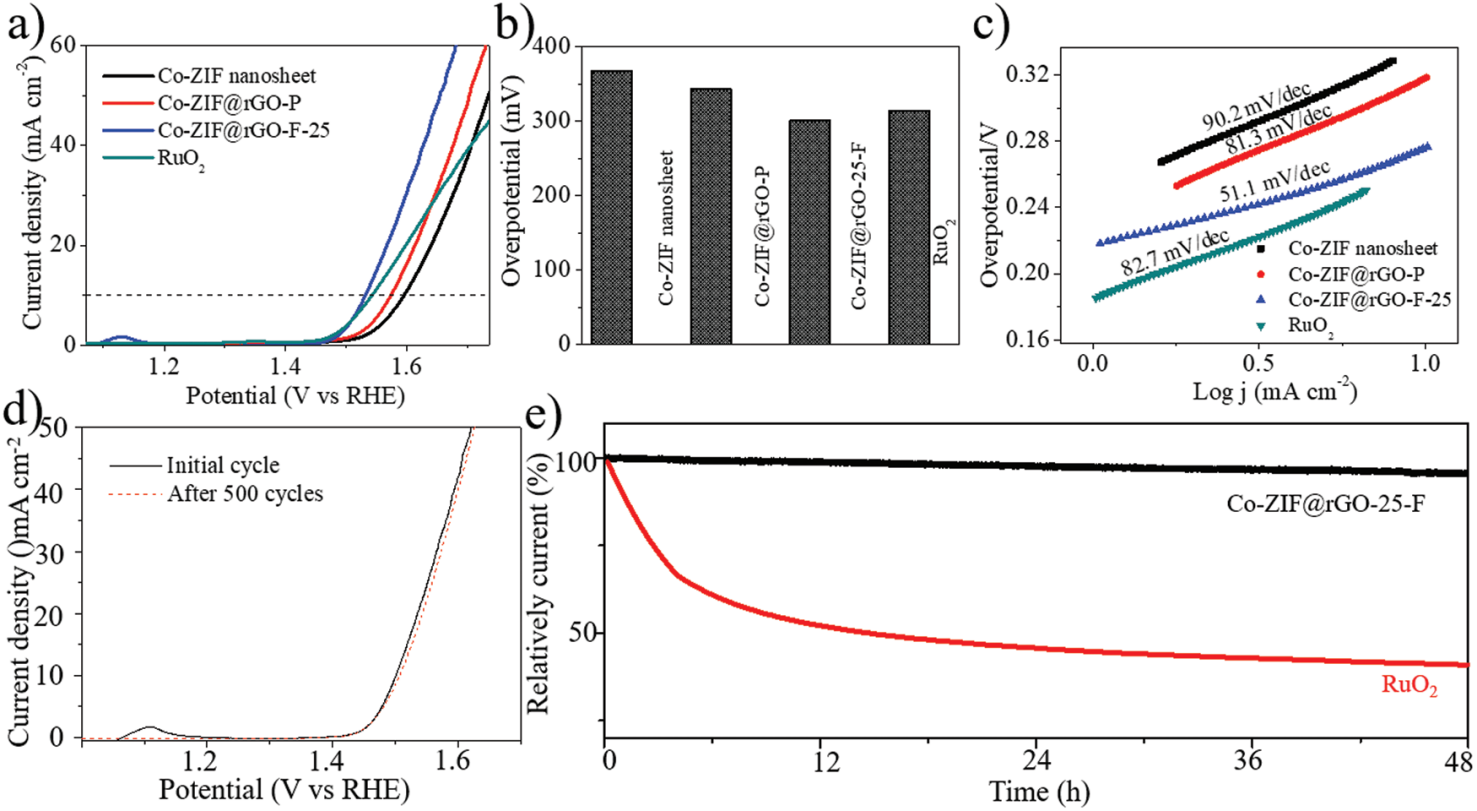
a) LSV plots, b) overpotentials required for *j* = 10 mA cm^−2^, c) Tafel slopes of Co‐ZIF@rGO‐F‐25, Co‐ZIF@rGO‐P, pure Co‐ZIF nanosheets and commercial RuO_2_ catalyst; d) LSV before and after successive CV scanning for 500 cycles at a scan rate of 10 mV s^−1^. e) Long‐term stability of Co‐ZIF@rGO‐F‐25 and RuO_2_.

Co‐ZIF@rGO‐F‐25 was also employed as electrode material for oxygen evolution reaction (OER) to further show the electrochemical advantages. Their performances were compared with Co‐ZIF@rGO‐P, pure Co‐ZIF nanosheets and a commercial RuO_2_ catalyst. The polarization curves recorded by linear sweep voltammetry (LSV) in 0.1 m KOH solution by using a three‐electrode system at a scan rate of 10 mV s^−1^ were presented in Figure 4. As shown in **Figure** **6**a,b, Co‐ZIF@rGO‐F‐25 possesses an obvious lower overpotential of 301 mV than those of Co‐ZIF@rGO‐P (343 mV), pure Co‐ZIF nanosheets (367 mV) and commercial RuO_2_ (314 mV). The tafel slope of Co‐ZIF@rGO‐F‐25 (51.1 mV dec^−1^) is also much smaller than the controlled samples including Co‐ZIF@rGO‐P (81.3 mV dec^−1^), pure Co‐ZIF nanosheets (90.2 mV dec^−1^) and commercial RuO_2_ (82.7 mV dec^−1^), respectively (Figure 6c). The OER performances of Co‐ZIF@rGO‐F‐25 are comparable with the reported best MOF‐based catalysts under the same conditions (Table S2, Supporting Information), indicating the superior OER activity of Co‐ZIF@rGO‐F‐25.

To evaluate the stability of Co‐ZIF@rGO‐F‐25, successive CV with a scan rate of 10 mV s^−1^ was performed. The LSV curve of Co‐ZIF@rGO‐F‐25 shows slightly increase of overpotential after 500 cycles (Figure 6d), revealing its robust catalytic activity. The long‐term durability of Co‐ZIF@rGO‐F‐25 was further measured by the chronoamperometric measurements. As shown in Figure 6e, the current decay of Co‐ZIF@rGO‐F‐25 is only ≈5.7% after 48 h. For comparison, the corresponding current loss at the RuO_2_ electrode is as high as 58.8%. The structure of Co‐ZIF@rGO‐F‐25 after cycling test was also characterized. The XRD pattern (Figure S33, Supporting Information) shows the transformation of Co‐ZIF into cobalt hydroxides and cobalt oxides during OER process, consistent with previous reports,^58^, ^59^ in which MOFs were also converted into metal oxides or hydroxides with stable OER activity. The FT‐IR spectra (Figure S34, Supporting Information) shows the typical peaks assigned to OH– and Co—O bond, in accordance with XRD result.

The OER activity of three other UMOFNs@rGO composites were also tested by LSV curves. As shown in Figure S35 (Supporting Information), the overpotentials of Ni‐ZIF@rGO‐F, Co‐ZIF@rGO‐F, Co, Cu‐ZIF@rGO‐F and Co, Fe‐ZIF@rGO‐F at current density of 10 mA cm^−2^ were measured to be 350, 301, 272, and 252 mV, respectively. The order of the activity is Co, Fe‐ZIF@rGO‐F > Co, Cu‐ZIF@rGO‐F > Co‐ZIF@rGO‐F > Ni‐ZIF@rGO‐F. The results indicate the introduction of Fe or Cu can enhance the OER activity of Co‐ZIF, consistent with the enhancement of OER activity in Co‐based catalysts in the presence of Fe or Cu.^60^, ^61^, ^62^

The remarkable electrochemical performance of Co‐ZIF@rGO‐F‐25 may be ascribed to the following reasons as shown in Figure 1b: 1) the relatively vertical growth direction is beneficial for anchoring more Co‐ZIF nanosheets on the substrate than the flat orientation, leading to higher Co‐ZIF content and more abundant active species; 2) the foam‐like interconnected Co‐ZIF nanosheets bring a well‐developed hierarchical network with rich open pores, which can efficiently promote the utilization of Co‐ZIF nanosheets, facilitate the diffusion of Li ions, electrolytes and O_2_ molecules and buffer the volume expansion; 3) the large interplanar spacing allows the Li ions to be quickly inserted into and extracted from; 4) the hybridization of Co‐ZIF nanosheets with rGO greatly facilitates the electron transfer and thus improves the conductivity and electrochemical stability. The remarkable performance of Co‐ZIF@rGO‐F‐25 for Li‐ion batteries and OER catalysis further demonstrates the structural advantages of 3D foam‐like MOFs nanosheet network.

## 3 Conclusion

In summary, ultrathin metal–organic framework nanosheets directly anchored on the surface of rGO with controllable orientations have been synthesized by a low‐concentration‐assisted heterogeneous‐nucleation process, which can be generally employed to construct various UMOFNs (e.g., Co‐ZIF, Ni‐ZIF, Co, Cu‐ZIF, Co, Fe‐ZIF)@rGO hybrids. The obtained UMOFNs@rGO‐F composites possess a foam‐like hierarchical structure with UMOFNs vertically attached on rGO, creating open pores and improved electrical conductivity. The application potential of UMOFNs@rGO‐F as electrode materials for Li‐ion batteries and oxygen evolution has been demonstrated, showing excellent performances compared to its counterparts. Our strategy has provided a new family of UMOFNs@rGO based hybrid materials with promising electrochemical applications.

## 4 Experimental Section

*Synthesis of Co‐ZIF@rGO‐F Hybrid Materials*: The GO was prepared according to a modified Hummer's method. For the synthesis of Co‐ZIF@rGO‐F, 0.3 mL of GO was sonicated in 6 mL methanol/water mixed solution (2:1 in volume). Then, 3 mL of 2‐MeIM solution (5, 10, 15, 25, and 50 × 10^−3^ m, respectively), 3 mL of Co(NO_3_)_2_·6H_2_O solution (5, 10, 15, 25, and 50 × 10^−3^ m, respectively) was sequentially added into above solution and then allowed to react at room temperature for 30 s without stirring, forming Co‐ZIF@GO‐F‐*x* suspensions. The product was then collected by centrifugation, washed with methanol for several times. Then, the obtained product was re‐dispersed into 20 mL of 15 × 10^−3^ m ascorbic acid solution and the mixture was further stirred at room temperature for 4 h. The final product was collected by centrifugation, washed with water several times, vacuum dried overnight and denoted as Co‐ZIF@rGO‐F‐*x*. The “*x*” denotes the concentration of 2‐MeIM solution and Co(NO_3_)_2_·6H_2_O solution.

## Conflict of Interest

The authors declare no conflict of interest.

## Supporting information

Supporting Information
