# Supplementary material for: A General Approach to Direct Growth of Oriented Metal–Organic Framework Nanosheets on Reduced Graphene Oxides
Source: Adv Sci (Weinh). 2020 Jan 3;7(4):1901480. doi: 10.1002/advs.201901480 (PMC7029658; doi:10.1002/advs.201901480)
Supplement: Supplementary file 1 — Supporting Information [file ADVS-7-1901480-s001.pdf]

Copyright WILEY-VCH Verlag GmbH & Co. KGaA, 69469 Weinheim, Germany, 2019.

ADVANCED SCIENCE
Open Access

Supporting Information

for *Adv. Sci.,* DOI: 10.1002/advs.201901480

A General Approach to Direct Growth of Oriented Metal–Organic Framework Nanosheets on Reduced Graphene Oxides

Chao Liu, Xiaodan Huang, Jizi Liu, Jing Wang, Zibin Chen, Rui Luo, Chaohai Wang, Jiansheng Li, Lianjun Wang, Jingjing Wan, and Chengzhong Yu**

Copyright WILEY-VCH Verlag GmbH & Co. KGaA, 69469 Weinheim, Germany, 2019.

Supporting Information

A General Approach to Direct Growth of Oriented Metal-Organic Framework Nanosheets on Reduced Graphene Oxides

Chao Liu,[1, 2, 3] Xiaodan Huang,[3] Jizi Liu,[4] Jing Wang,[1, 2, 3] Zibin Chen,[5] Rui Luo,[2] Chaohai Wang,[2] Jiansheng Li,[2] Lianjun Wang [2], Jingjing Wan[1] and Chengzhong Yu*[1, 3]*

Chemicals. Cobalt nitrate·6H_2O (98 %, Aldrich), cupper nitrate·3H_2O (98 %, Aldrich), ferrous sulfate·7H_2O (99 %, Aldrich), 2-methylimidazole (2-MeIM, Aldrich), hydrazine hydrate (Aldrich), graphite (99.8 %, Alfa Aesar), methanol (AR), Millipore water was used in all experiments.

Synthesis of Co-ZIF nanosheets. The typical synthesis process of Co-ZIF nanosheets was as follows: 3 mL of 25 mM 2-MeIM solution, 3 mL of 25 mM $Co(NO_3)_2 \cdot 6H_2O$ solution was sequentially added into 6 mL methanol/water mixed solution and then allowed to react at room temperature for 30 s without stirring. The product was collected by centrifugation, washed with water several times and vacuum dried overnight.

Synthesis of Co-ZIF@rGO-P hybrid materials. The synthesis procedure of Co-ZIF@rGO-P was similar to Co-ZIF@rGO-F except pre-reducing GO to rGO by ascorbic acid before growing Co-ZIF nanosheets.

Synthesis of Ni-ZIF@rGO-F hybrid materials. The synthesis procedure of Ni-ZIF@rGO-F was similar to Co-ZIF@rGO-F except using $Ni(NO_3)_2 \cdot 6H_2O$ (25 mM) as percursor.

Synthesis of Co,Cu-ZIF@rGO-F hybrid materials. The synthesis procedure of Co,Cu-ZIF@rGO-F was similar to Co-ZIF@rGO-F except using $Co(NO_3)_2 \cdot 6H_2O$ (25 mM) and $Cu(NO_3)_2 \cdot 3H_2O$ (2.5 mM) as percursors.

Synthesis of Co,Fe-ZIF@rGO-F hybrid materials. The synthesis procedure of Co,Fe-ZIF@rGO-F was similar to Co-ZIF@rGO-F except using $Co(NO_3)_2·6H_2O$ (25 mM) and $FeSO_4·7H_2O$ (2.5 mM) as percursors.

Synthesis of *a*Co(OH)$_2$: *a*$Co(OH)_2$ was synthesized according to a previous report.[50] The synthesis was conducted under N_2 atmosphere. 0.56 mmol of the total amount of $Co(NO_3)_2$•$6H_2O$, $NaNO_3$ (0.5 M, 2 mL), NH_4F (0.5 M, 6mL) and sodium citrate (0.01M, 2ml) were dispersed in 100 mL H_2O (purged with N_2). The pH of the solution was then tuned to ~12 by drop-wise addition of NaOH solution (0.08 M, 40 mL, purged with N_2 for 30 min). Then the resulting suspension was aged at room temperature for 18 h. The solid product was isolated by centrifugation, washed and dried under ambient conditions.

Material characterization. Transmission electron microscopy (TEM) images were obtained with Hitachi HT7700 at 120 KV. High resolution transmission electron microscopy analyses were conducted on a FEI Tecnai F30 G2 operated at 300 kV at low electron dose (~1 electron per square Å). X-ray diffraction (XRD) patterns were recorded using a Bruker D8 Advanced X-Ray Diffractometer with Co Kα radiation (λ=0.179 nm). Fourier transform infrared (FT-IR) spectra of samples were obtained by using FT-IR-Nicolet IS-10 Thermo Fisher. To determine the contents of Co-ZIF in samples, all samples were digested by dilute nitric acid solution, then the mixture were filtered through 0.22 mm membrane filter and the metal ion containing filtrates were analyzed by inductive coupled plasma atomic emission spectroscopy (ICP-AES) (Optima 7000DV, PerkinElmer, USA). The height and thickness of samples were analyzed by an atomic force microscopy (AFM, Dimen-sion Icon, Bruker, USA). The XPS spectra were obtained by using a PHI Quantera II ESCA System with Al Kα radiation at 1486.8 V. UV-vis spectra were obtained by using a UV-Vis spectrophotometer (Perkin Elmer Lambda 750). The N_2 sorption test was performed on a ASAP 2460 intrusment with degassing time of 24 h.

Li-ion batteries test. The electrochemical properties of Co-ZIF@rGO-F-25 were measured using coin-type half cells (2016 R-type) which assemble under an argon-filled glove box (H_2O, O_2<0.1 ppm). The working electrode was prepared by mixing active composites, super P carbon black and poly(vinylidene fluoride), with a weight ratio of 80 : 10 : 10. The slurry was pasted on a Cu foil and then dried in a vacuum oven at 60 °C for 15 h. The active material density of each cell was determined to be 1.1-1.3 mg cm^{-2}. A metallic Li sheet was used as the counter electrode, and LiPF6 (1 M) in EC-DMC-EMC (1:1:1 vol %) was used as the electrolyte. The charge-discharge performance was tested between 0.01 and 3.00 V using were performed on a Land 8 Channel Battery Analyzer at room temperature. The capacities of electrodes were determined using the total mass of Co-ZIF@rGO composites. The electrochemical impedance spectroscopy (EIS, frequency ranging from 10^6 Hz to 10^{-2} Hz with an AC amplitude of 5 mV) were carried out with the cells using a CHI660e electrochemical workstation (ChenHua Instruments Co., China).

OER test. OER was studied in a standard three-electrode system with Co-ZIF@rGO-F-25 as the working electrode, platinum wire as a counter electrode and Ag/AgCl as a reference electrode in a CHI 660E electrochemistry workstation (ChenHua Instruments Co., China). All the potentials in our measurements were transformed to reversible hydrogen electrodes (RHE) according to Potential=EAg/AgCl + 0.059 pH + 0.197 V. The working electrode was fabricated as follows: 5.0 mg catalyst was dispersed in 0.95 mL of ethanol and 0.05 mL of 5.0 wt.% Nafion solution to generate a relatively homogeneous suspension. Then the suspension (10 μL) was loaded onto the glass carbon disk electrode (0.196 cm^2). Linear sweep voltammograms (LSV) were acquired through the rotating disk electrode (RDE) technique in 0.1 M KOH at a scan rate of 10 mV s-1. The Tafel slope is calculated according to the equation as follows: $\eta = a + b \log j$, η means the overpotential, a means contant, b maens the Tafel slope, and j means the current density. Long-term stability tests were performed by

chronoamperometric measurement at the overpotential of 301 mV and continuous CV scans in the range of 1 to 1.7 V versus RHE at a sweep rate of 20 mV s^{-1}.

Li-ion batteries test. The electrochemical properties of Co-ZIF@rGO-F-25 were measured using coin-type half cells (2016 R-type) which assemble under an argon-filled glove box (H_2O, O_2<0.1 ppm). The working electrode was prepared by mixing active composites, super P carbon black and poly(vinylidene fluoride), with a weight ratio of 80 : 10 : 10. The slurry was pasted on a Cu foil and then dried in a vacuum oven at 60 °C for 15 h. The active material density of each cell was determined to be 1.1-1.3 mg cm^{-2}. A metallic Li sheet was used as the counter electrode, and LiPF6 (1 M) in EC-DMC-EMC (1:1:1 vol %) was used as the electrolyte. The charge-discharge performance was tested between 0.01 and 3.00 V using were performed on a Land 8 Channel Battery Analyzer at room temperature. The capacities of electrodes were determined using the total mass of Co-ZIF@rGO composites. The electrochemical impedance spectroscopy (EIS, frequency ranging from 10^6 Hz to 10^{-2} Hz with an AC amplitude of 5 mV) were carried out with the cells using a CHI660e electrochemical workstation (ChenHua Instruments Co., China).

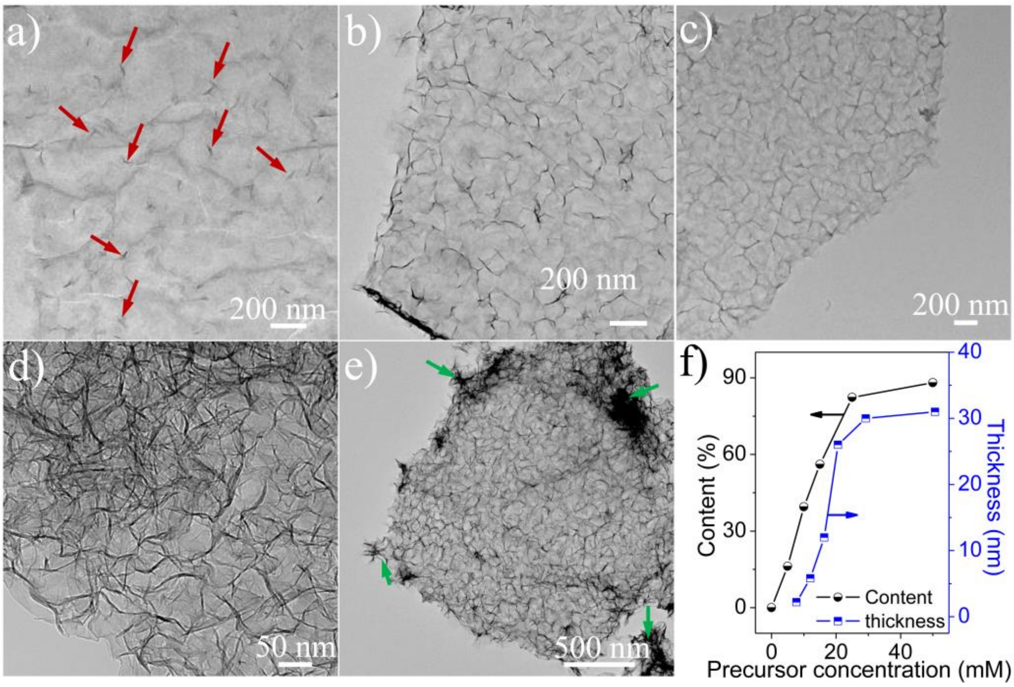

Figure S1. TEM images of (a) Co-ZIF@rGO-F-5, (b) Co-ZIF@rGO-F-10, (c) Co-ZIF@rGO-F-15, (d) Co-ZIF@rGO-F-25, (e) Co-ZIF@rGO-F-50; (f) The content (from ICP measurements) of Co-ZIF nanosheets in Co-ZIF@rGO-F-x samples and thickness (obtained from Figure S2) of Co-ZIF@rGO-F-x.

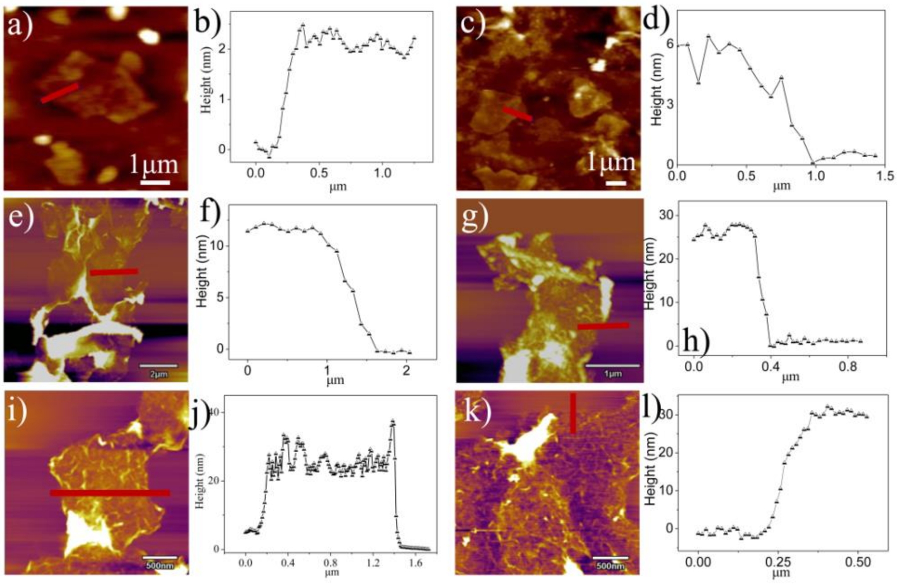

Figure S2. AFM images and height profiles of (a, b) rGO, (c, d) Co-ZIF@rGO-F-5, (e, f) Co-ZIF@rGO-F-10, (g, h) Co-ZIF@rGO-F-15, (i, j) Co-ZIF@rGO-F-25, (k, l) Co-ZIF@rGO-F-50.

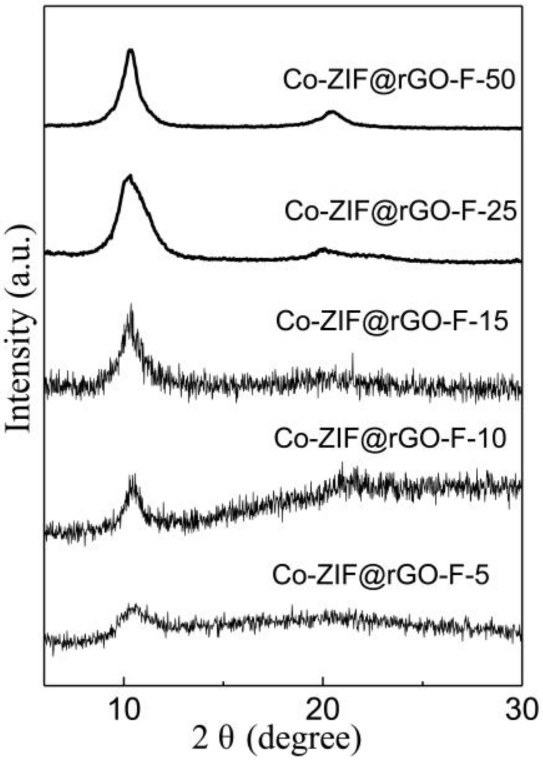

Figure S3. XRD patterns of Co-ZIF@rGO-F-x.

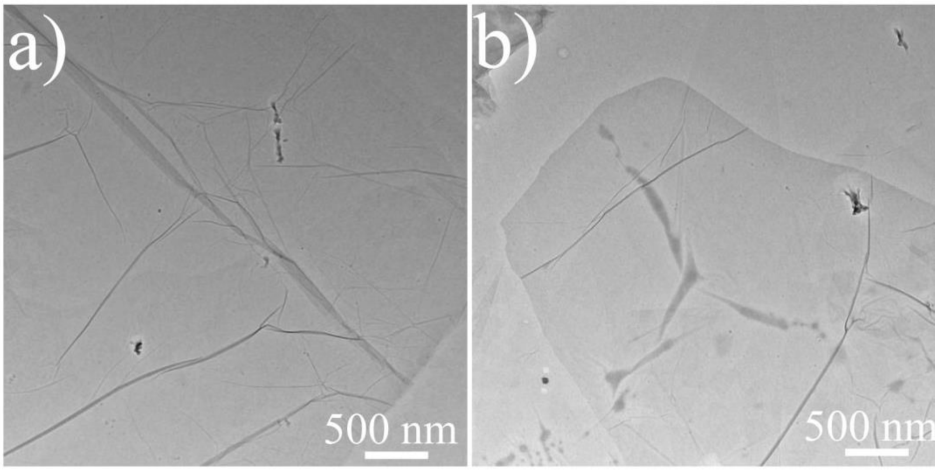

Figure S4. TEM images of (a) GO, (b) rGO.

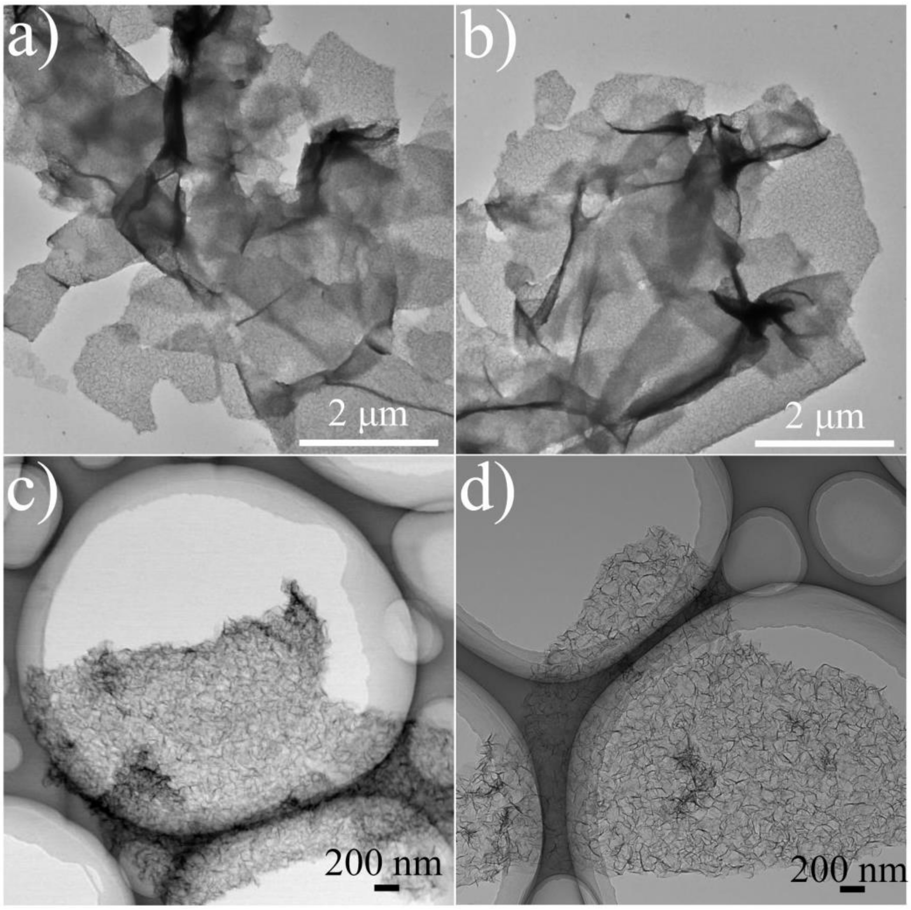

Figure S5. TEM images of Co-ZIF@rGO-F-25 in different areas.

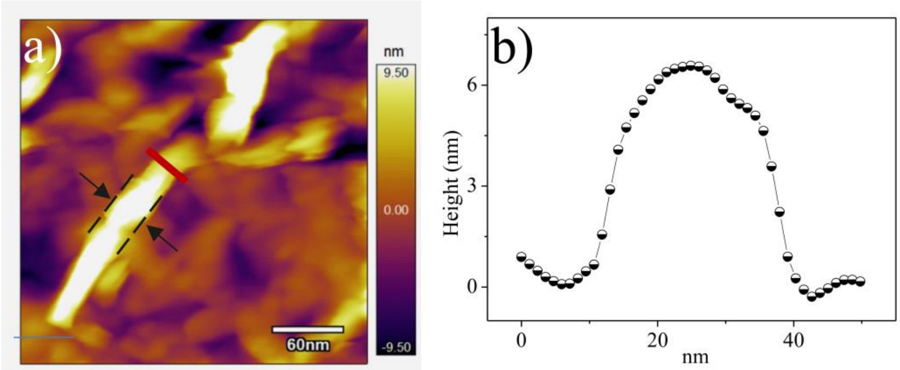

Figure S6. (a) AFM images of Co-ZIF@rGO-F-25; (b) The thickness profile of Co-ZIF nanosheets on Co-ZIF@rGO-F-25.

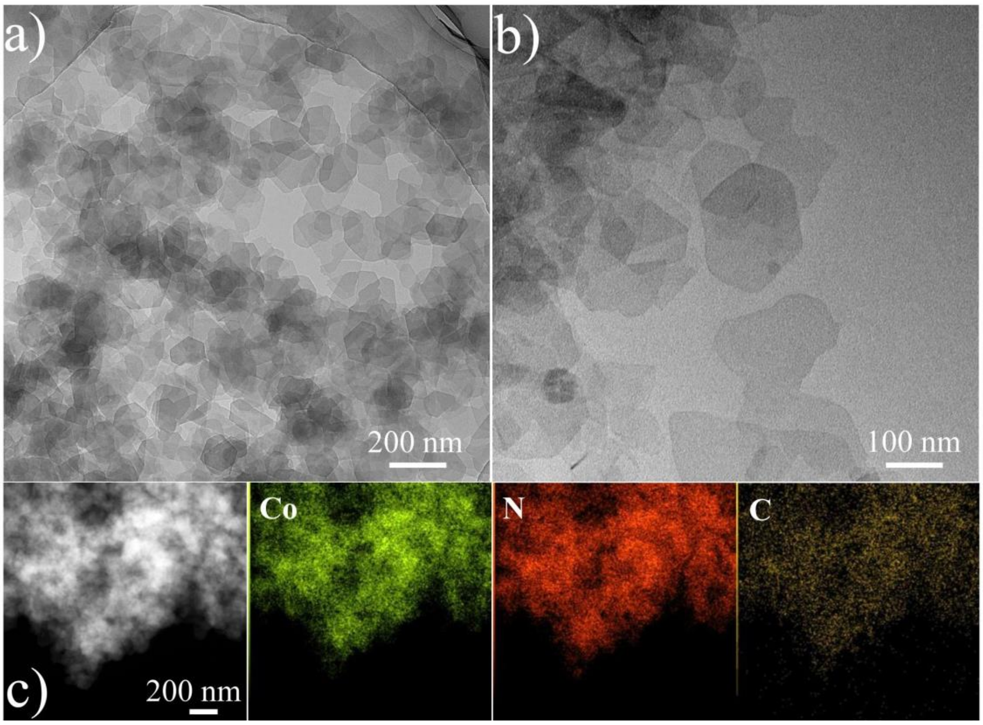

Figure S7. (a, b) TEM images, (c) STEM image and element mapping of Co-ZIF nanosheets. The presence and uniform distribution of Co, N, and C in the Co-ZIF nanosheets is evidenced by the corresponding elemental mapping, in agreement with the result of Co-ZIF@rGO-F.

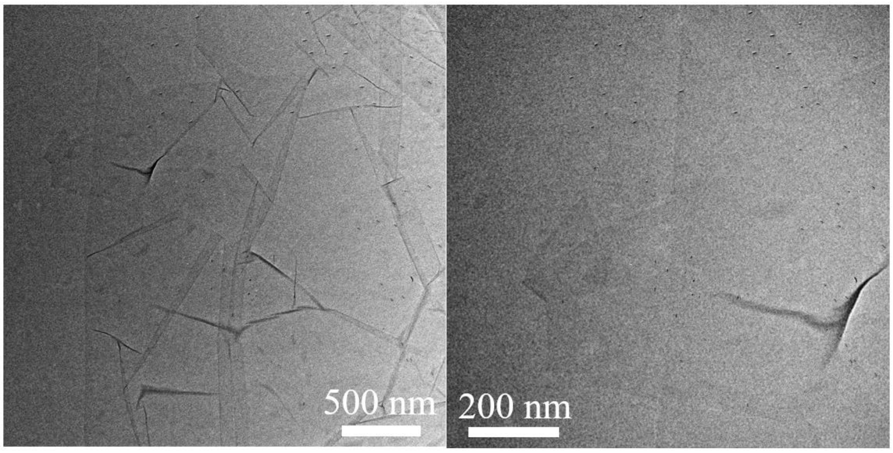

Figure S8. TEM image of rGO obtained by removing the Co-ZIF part in Co-ZIF@rGO-F-25 using 1M HCl.

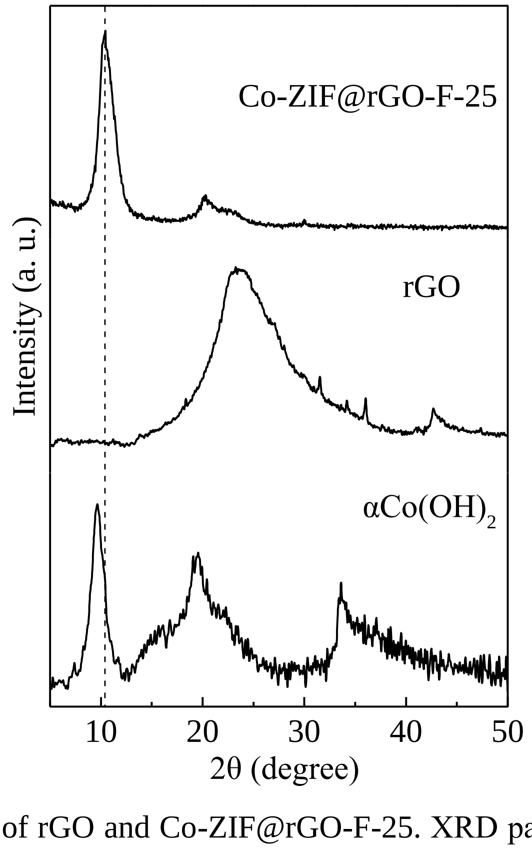

Figure S9. XRD patterns of rGO and Co-ZIF@rGO-F-25. XRD patterns of α-$Co(OH)_2$, rGO and Co-ZIF@rGO-F-25. The XRD pattern of synthesized α-$Co(OH)_2$ displays three major diffraction peaks at 9.6, 19.2 and 33.7^o, corresponding to the (003), (006) and (009) facets for hydrotalcite-like material (JCPDS No.51-0916), in agreement with literature reports. The two diffractions of Co-ZIF@rGO-F-25 are located at 10.4 and 20.8^o, different from α-$Co(OH)_2$ or

rGO which has a broad diffraction centered at 23-24°. Thus, the diffraction peaks observed in Co-ZIF@rGO-F-25 should be assigned to the Co-ZIF, rather than rGO nor α-$Co(OH)_2$.

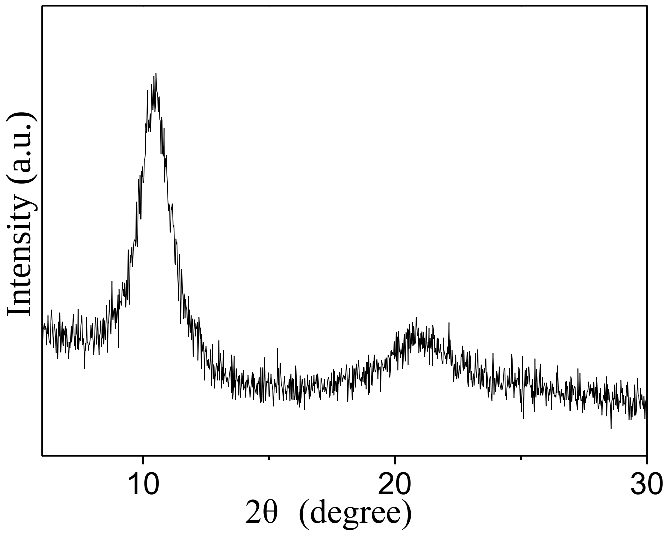

Figure S10. XRD pattern of Co-ZIF@rGO-25-F prepared using the reported acetone-assisted method. Typically, 2-time volume of acetone was poured into the methanol suspension of Co-ZIF@rGO-25-F.

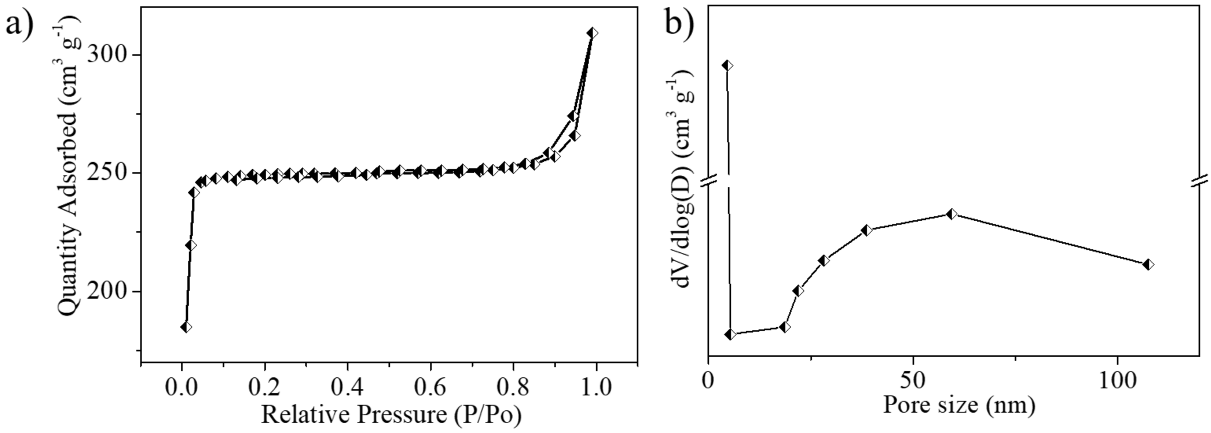

Figure S11. (a) N_2 sorption curves and (b) pore size distribution of Co-ZIF@rGO-F-25. The BET surface area of Co-ZIF@rGO-F-25 is measured to be 762.3 m2 g^{-1}. The pore size distribution curve shows a wide distribution in the range of 18.2-117.6 nm, indicating the hierarchical porous structure.

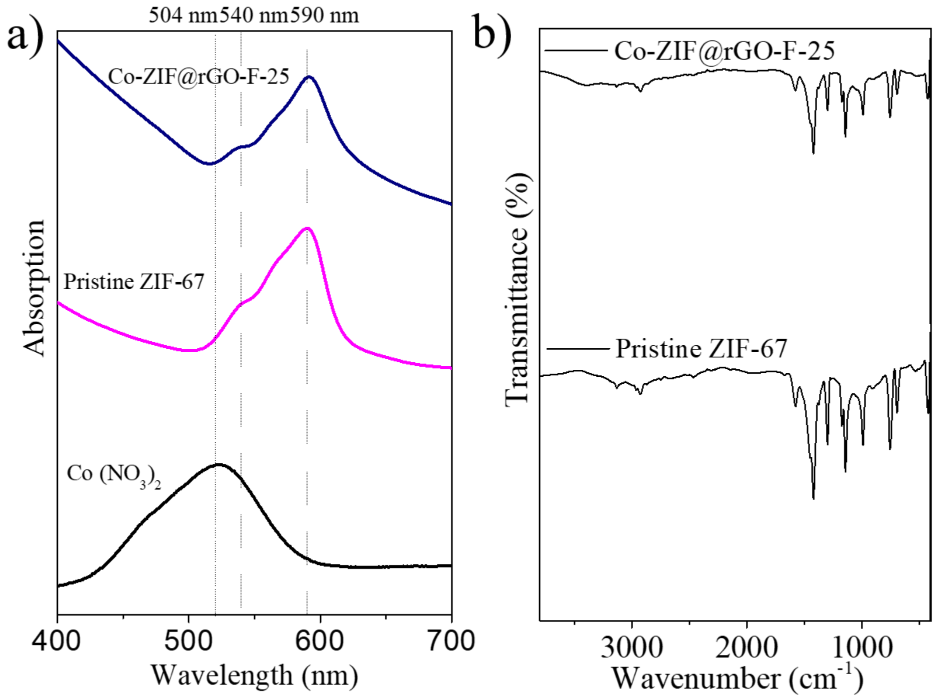

Figure S12. (a) UV-vis spectra of $Co(NO_3)_2$, pristine ZIF-67 and Co-ZIF@rGO-F-25, (b) FT-IR spectra of pristine ZIF-67 and Co-ZIF@rGO-F-25.

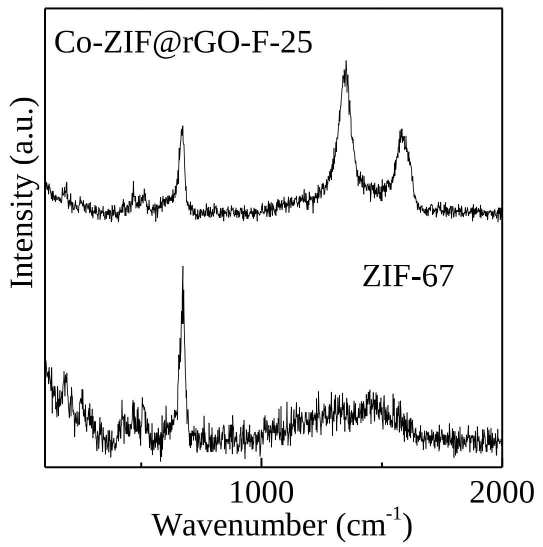

Figure S13. Raman spectra of pristine ZIF-67 and Co-ZIF@rGO-F-25.

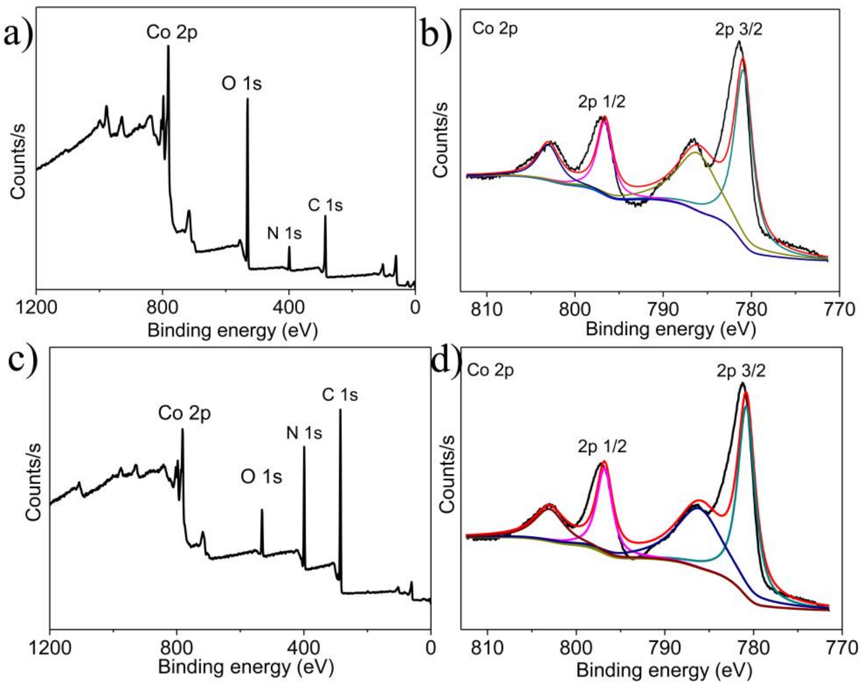

Figure S14. XPS survey spectrum of (a) Co-ZIF@rGO-F-25, (c) pristine ZIF-67; high-resolution XPS Co 2p spectrum of (b) Co-ZIF@rGO-F-25, (d) pristine ZIF-67. The peaks, corresponding to Co, O, N, C, are observed in Figure S S10a. Two main peaks at 780.9 and 796.6 eV in the high-resolution spectrum of Co 2p (Figure S S10b) are assigned to Co 2p 3/2and Co 2p 1/2, respectively, in agreement with the pristine ZIF-67 particles.

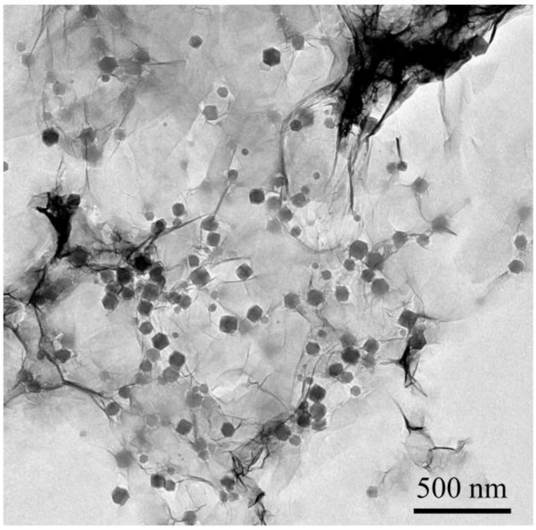

Figure S15. TEM image of ZIF-67@GO composite, which was synthesized by adding 2-MeIM and Co^{2+} (500 mM) into GO solution.

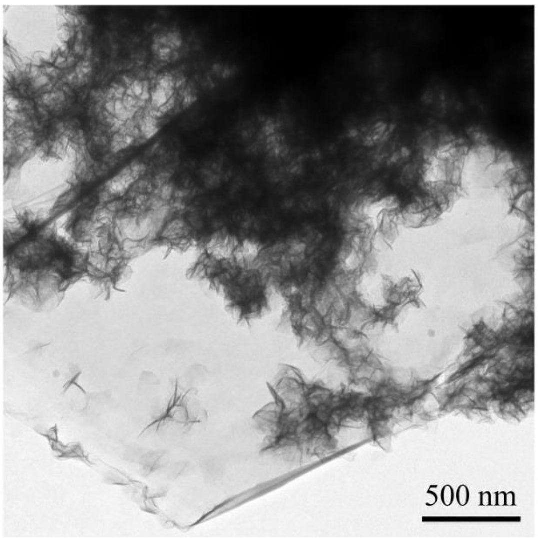

Figure S16. TEM image of ZIF-67@GO composite, which was synthesized by adding 2-MeIM and Co^{2+} (25 mM) into the solution before the addition of GO.

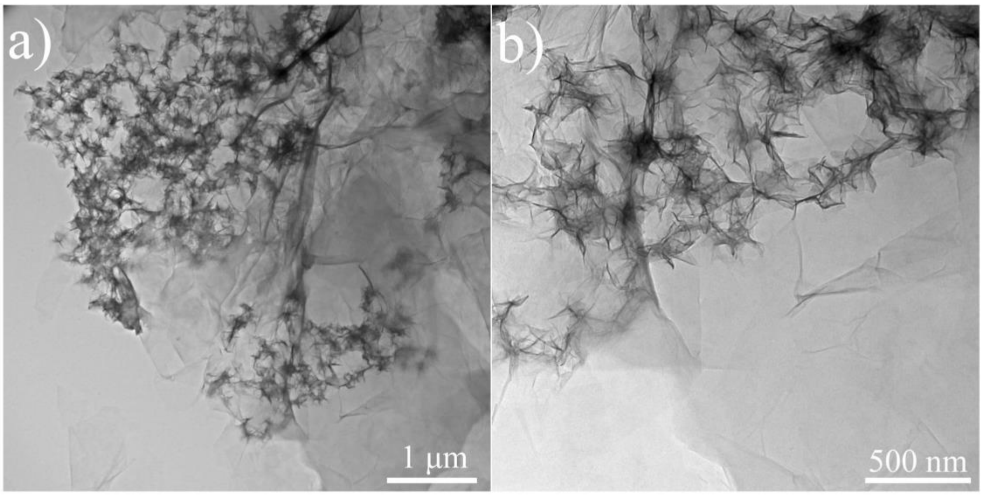

Figure S17. TEM images of ZIF-67@rGo composites synthesized at conditions similar to that of Co-ZIF@rGO-F-25, except adding Co^{2+} first followed by 2-MeIM.

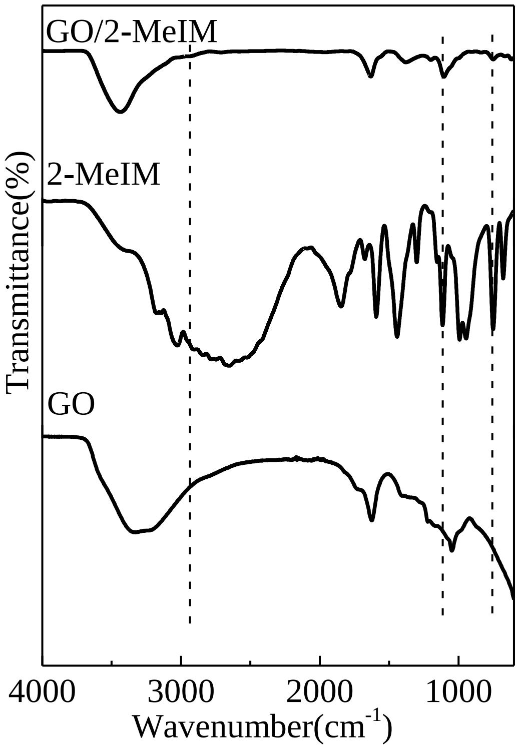

Figure S18. FT-IR spectra of GO, 2-MeIM and GO/2-MeIM.

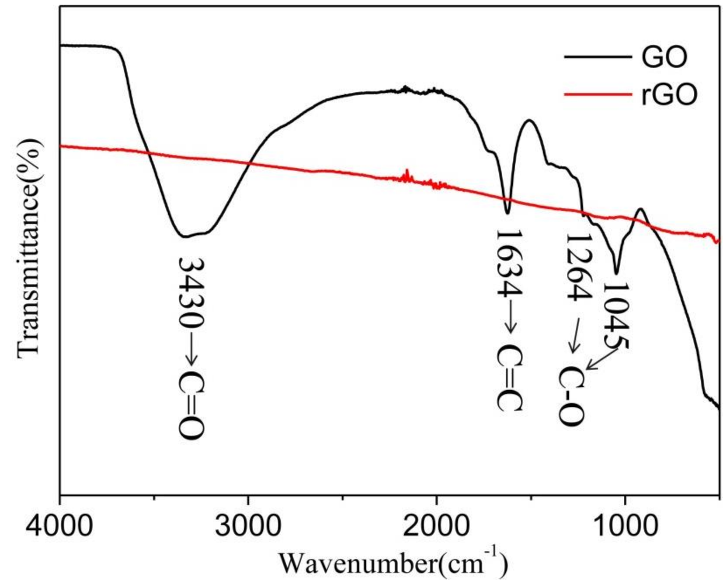

Figure S19. FTIR spectrums of GO and rGO.

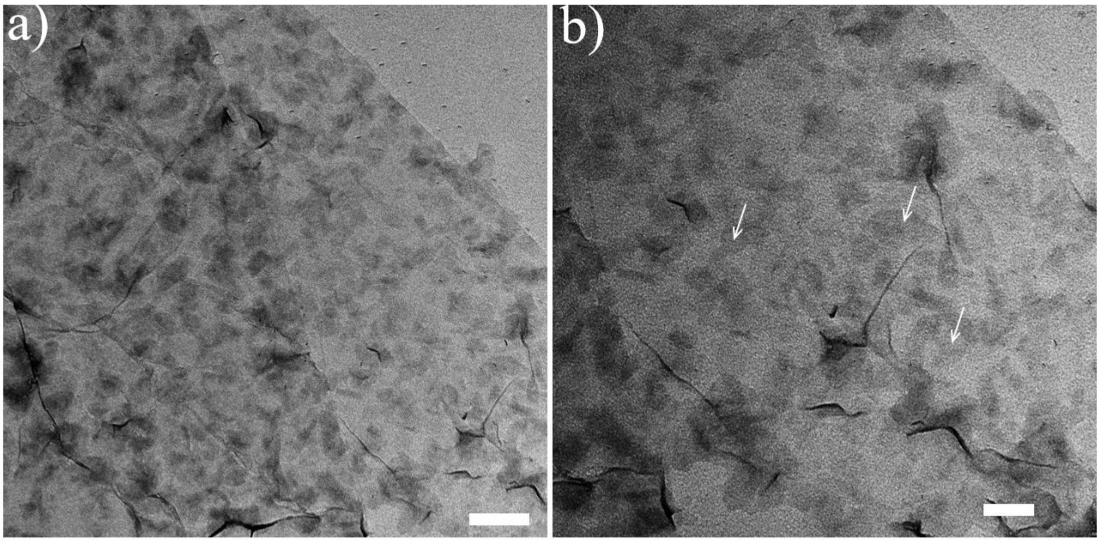

Figure S20. Enlarged TEM images of Co-ZIF@rGO-P.

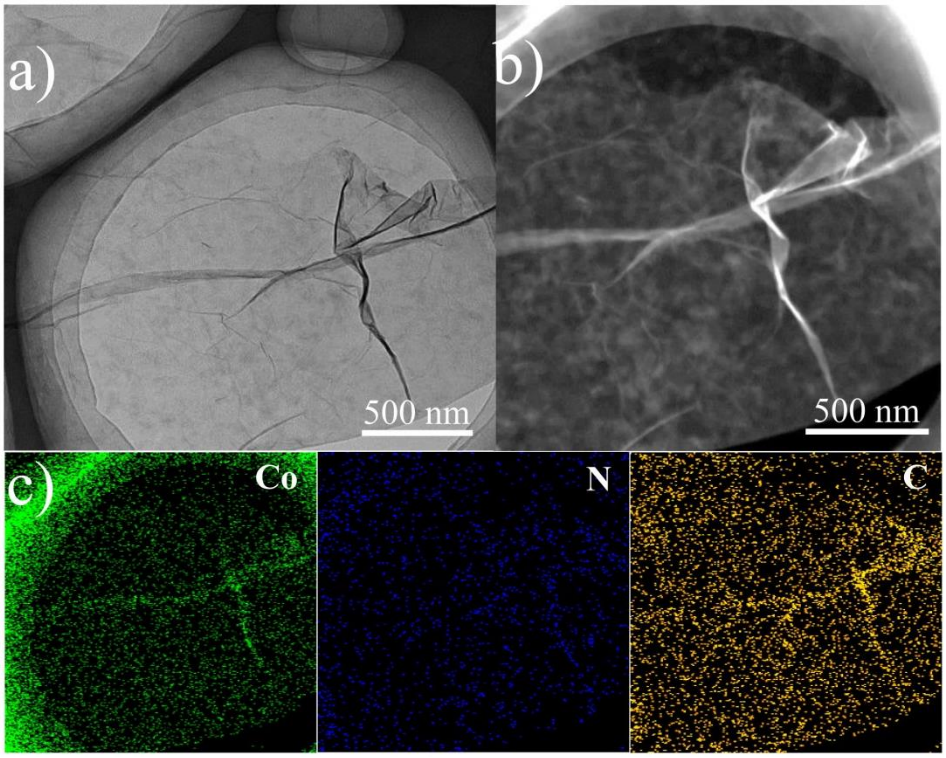

Figure S21. (a) TEM images, (b, c) STEM image and element mapping of Co-ZIF@rGO-P hybrids. STEM image shows that a large amount of nano-sized species are parallelly anchored on rGO without specific direction. The images of element mapping demonstrate the uniform distribution of Co, N and C.

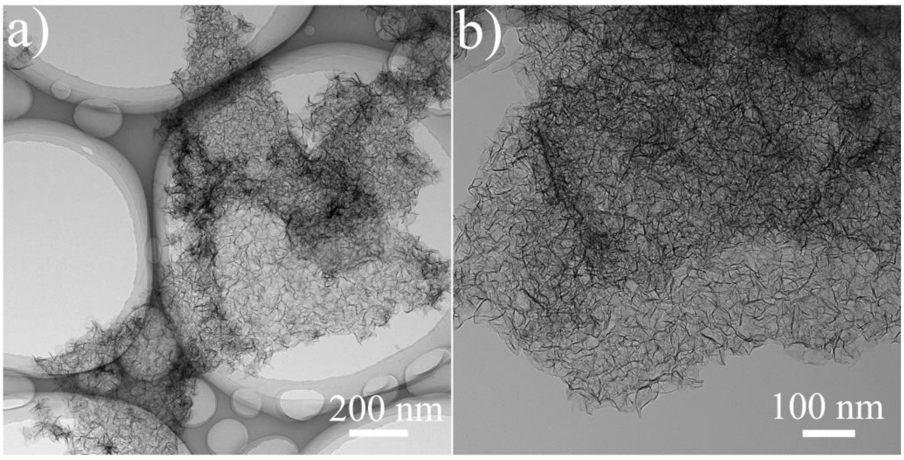

Figure S22. TEM images of freeze-dried Co-ZIF@GO-F-25. After reaction of 30 seconds, a TEM copper grid was dipped into the reaction solution and sealed in a 1 mL centrifugation tube. Then the tube was immersed in liquid nitrogen for 5 minute. The tube with the grid was

freeze-dried in a freeze drying equipment (FD-1-50, Beijing Boyikang Instruments Co., China) overnight before TEM characterization.

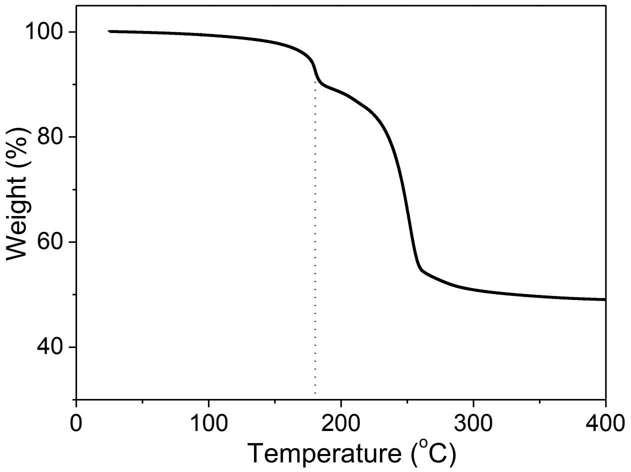

Figure S23. TGA curve of Co-ZIF@rGO-F-25 in air. The ramp rate was 5 ^{o}C/min. Based on this weight loss and the corresponding decomposition process, the content of Co-ZIF is then calculated to be ~76%, slightly lower than that measured by ICP (~82.3%).

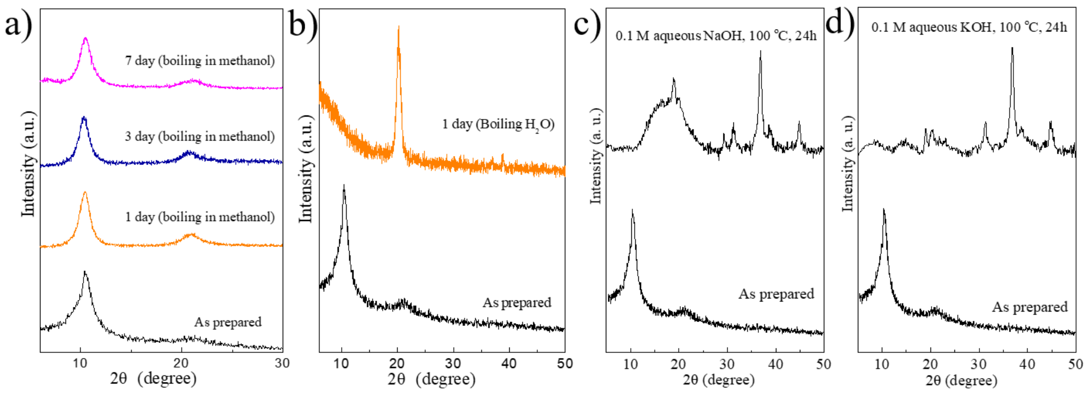

Figure S24. XRD patterns of Co-ZIF@rGO-F-25 samples measured during chemical stability tests. (a) In refluxing methanol at 65°C for up to 7 days. (b) In refluxing water at 100°C for up to 1 day. In refluxing aqueous (c) NaOH and (d) KOH solution for up to 1 day.

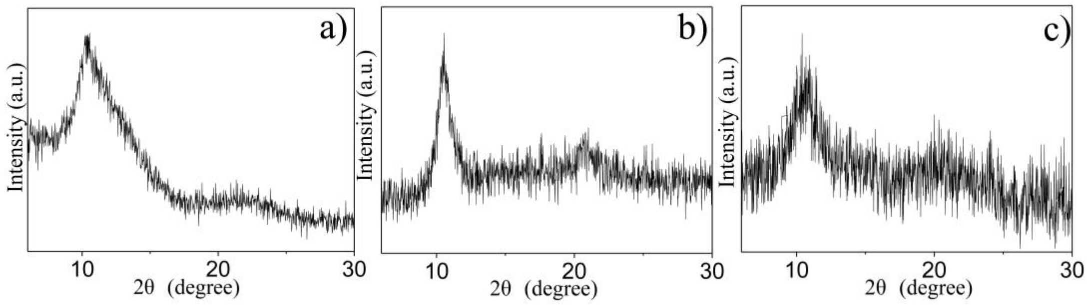

Figure S25. XRD patterns of (a) Ni-ZIF@rGO-F, (b) Co,Cu-ZIF@rGO-F, (c) Co, Fe-ZIF@rGO-F.

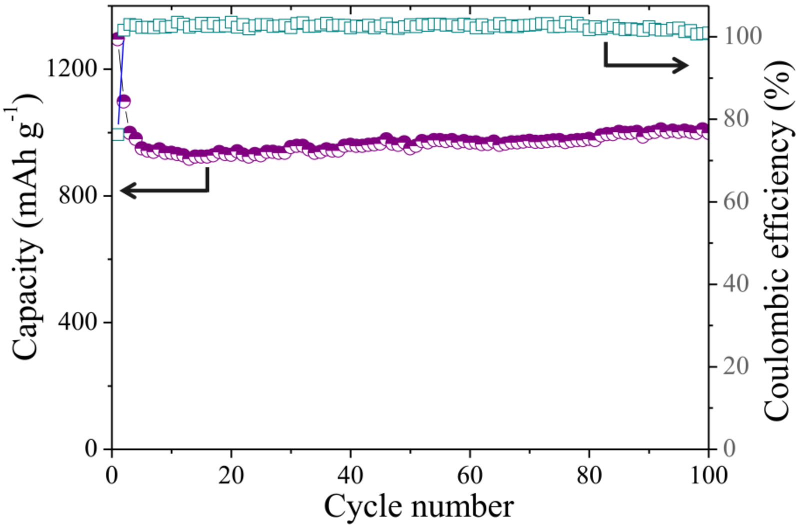

Figure S26. The cycling performances of Co-ZIF@rGO-F-25 with a loading mass of 2.35 mg/cm^2 at a current density of 0.1 A g^{-1}.

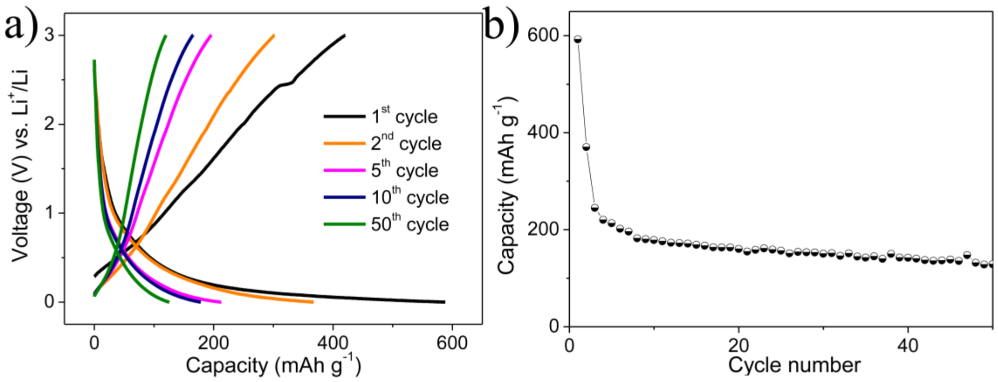

Figure S27. (a) The representative galvanostatic charge-discharge profiles and (b) cycling performance of pristine reduced graphene oxide at a current density of 0.1 A g^{-1}.

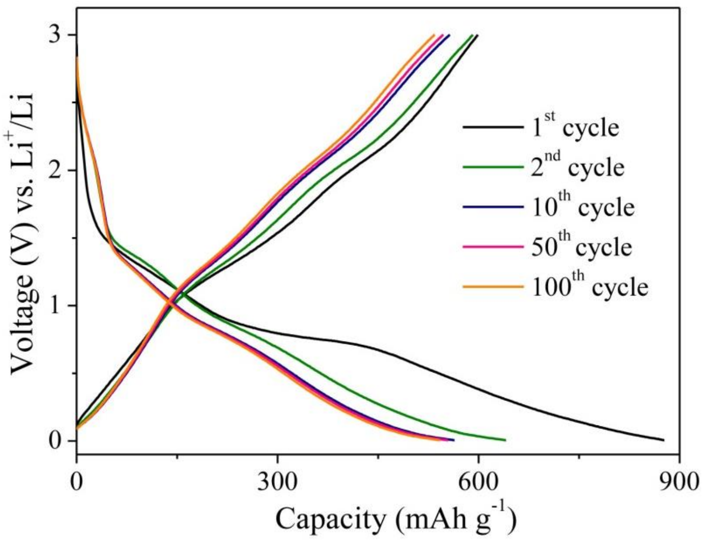

Figure S28. The representative galvanostatic charge-discharge profiles of Co-ZIF@rGO-P at a current density of 0.1 A g^{-1}.

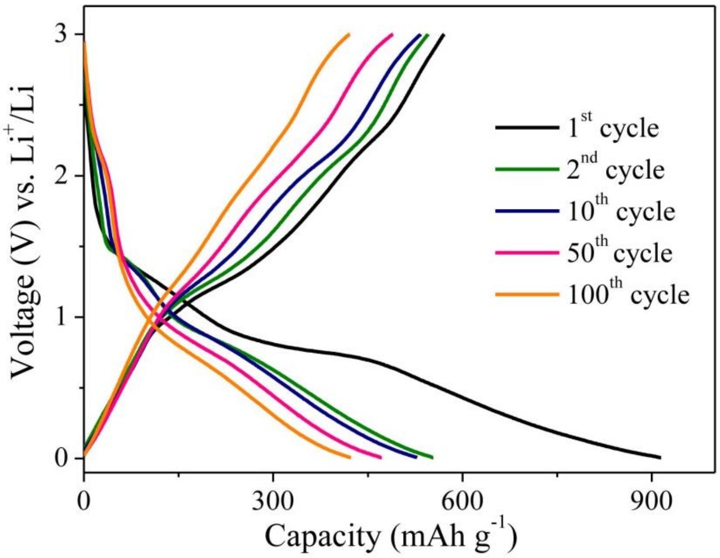

Figure S29. The representative galvanostatic charge-discharge profiles of Co-ZIF nanosheet at a current density of 0.1 A g^{-1}.

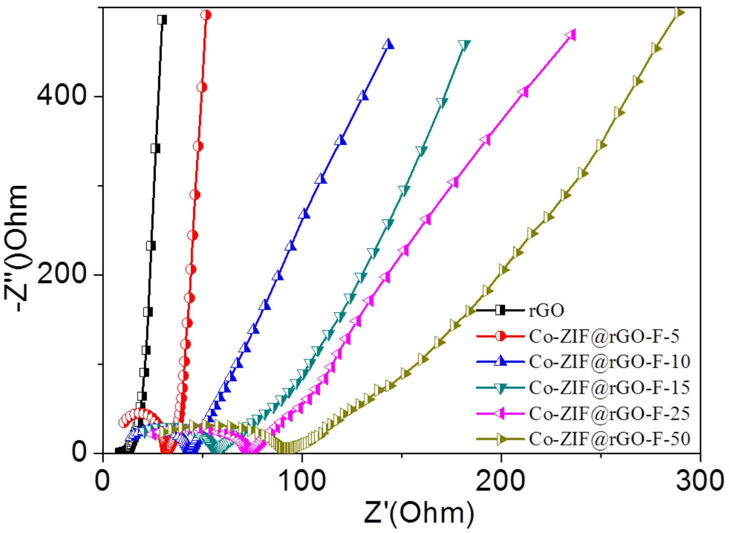

Figure S30. Electrochemical impedance spectroscopy of rGO and Co-ZIF@rGO-F-x.

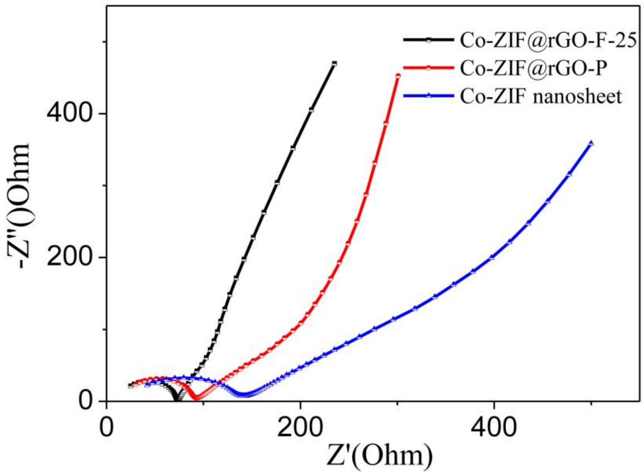

Figure S31. Electrochemical impedance spectroscopy of Co-ZIF nanosheets, Co-ZIF@rGO-P and Co-ZIF@rGO-F-25.

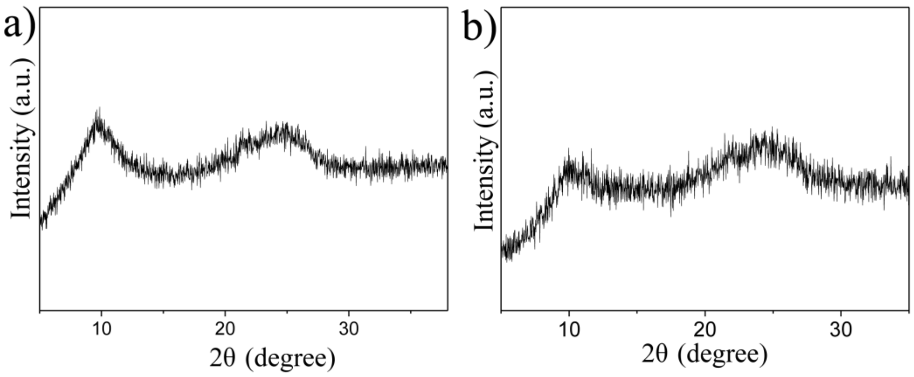

Figure S32. The XRD pattern of Co-ZIF@rGO-P and Co-ZIF nanosheets based anodes after cycling test.

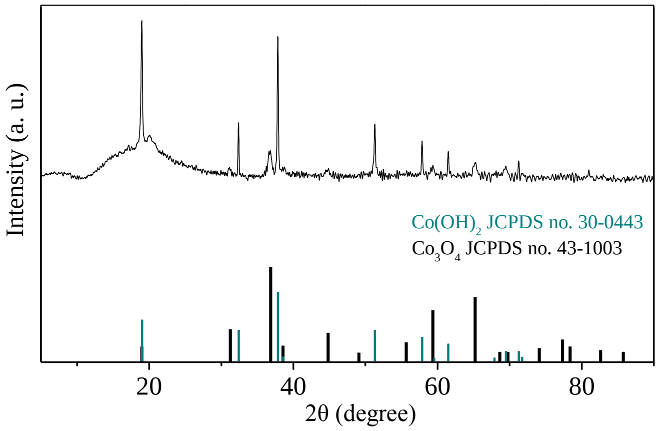

Figure S33. XRD pattern of Co-ZIF@rGO-F-25 after OER reaction.

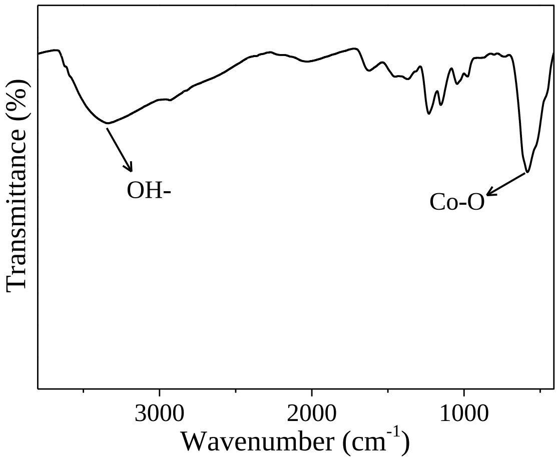

Figure S34. FT-IR spectrum of Co-ZIF@rGO-F-25 after OER reaction.

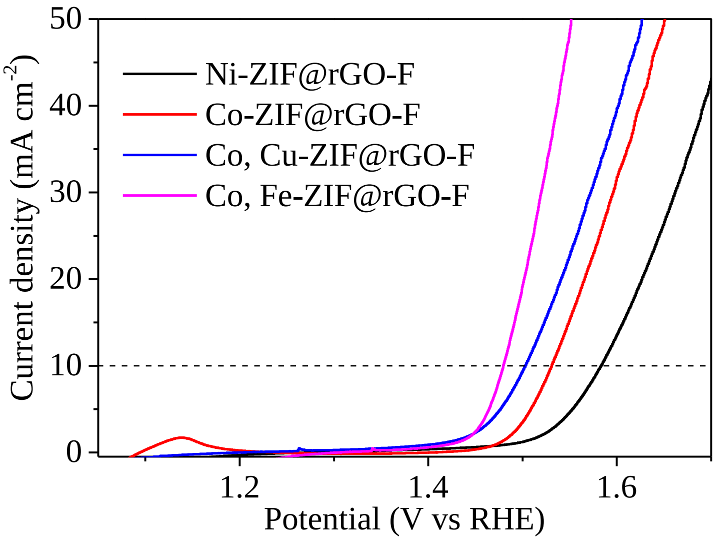

Figure S35. The LSV curves of different UMOFNs@rGO hybrids.

Table S1. Electrochemical performance of typical MOFs as anodes for Li-ions batteries

Materials	Current rate (A g^{-1})	Capacity (mAh g^{-1})	Initial columbic efficiency	References
Mn-LCP	0.05<br>390 (after 50 cycles)		30.5 %	S1
$Li_2C_8H_4O_4$	0.30<br>234 (after 50 cycles)			S2
BMOF	0.10<br>190 (after 200 cycles)		~55 %	S3
$[Co(H_2O)_6][Co_6(bpybdc)_2(N_3)10(H_2O)_4]\cdot 8H_2O$	0.103<br>694 (after 100 cycles)		40 %	S4
Co(L) MOF/RGO	0.1<br>1185 (after 150 cycles)	0.5<br>639 (after 120 cycles)	54.5 %	S5
Co-Zn-MOF	0.10<br>1211 (after 100 cycles)	1.0<br>753 (after 500 cycles)	64.08 %	S6
$Co_2(OH)_2BDC$	0.10<br>235.9 (after 20 cycles)	1.0<br>132.5 (after 20 cycles)	52 %	S7
CoBTC	0.10<br>856 (after 100 cycles)		49.15 %	S8
POMOF/RGO	0. 10<br>906 (after 10 cycles)		64.1 %	S9
Co-ZIF@rGO-F-25[a]	0.10<br>1024.1 (after 100 cycles)	1.0<br>713.2 (after 100 cycles)	71.0 %	This work

Table S2. Comparison of OER activity of Co-ZIF@rGO-F-25 and recently reported MOF-based active catalysts in 0.1 M KOH solutions.

**Sample**	**Overpotential/ mV (at 10 mA cm^{-2})**	**Tafel slope /mV dec^{-1}**	**Ref.**
Co-BDC/$Ti_3C_2T_x$	410	48.2	S10
MOF derived carbon nanocage	330	51	S11
CoFe-PYZ	300	44	S12
NNU-23	365	77.2	S13
Ni-UMOFNs	321	65	S14
Co-MOF	320	142	S15
ZIF-67 derived hollow framework	380	93	S16
NiCo-UMOFNs	250	42	S14
FeTPyP-Co	351	-	S17
Co-UMOFNs	371	103	S14
Co-ZIF-9	510@1mA cm^{-2}	93	S18
MAF-X27-OH	387	60	S19
Co-ZIF@rGO-F-25	301	51.1	This work

References

[1] Liu, Q. *et al.* Manganese-based layered coordination polymer: synthesis, structural characterization, magnetic property, and electrochemical performance in lithium-ion batteries. Inorg. Chem. **2013**, *52*, 2817.

[2] Armand, M. *et al.* Conjugated dicarboxylate anodes for Li-ion batteries. Nat. Mater. **2009**, *8*, 120.

[3] Lin, Y. *et al.* An exceptionally stable functionalized metal-organic framework for lithium storage. Chem. Commun. **2015**, *51*, 697.

[4] Maiti, S., Pramanik, A., Manju, U., Mahanty, S. Reversible Lithium Storage in Manganese 1,3,5-Benzenetricarboxylate Metal−Organic Framework with High Capacity and Rate Performance. ACS Appl. Mater. Interfaces **2015**, *7*, 16357.

[5] Dong, C., Xu, L. Cobalt- and cadmium-based metal-organic frameworks as high-performance anodes for sodium ion batteries and lithium ion batteries. ACS Appl. Mater. Interfaces **2017**, *9*, 7160.

[6] Li, C., Hu, X., Lou, X., Chen, Q., Hu, B. Bimetallic coordination polymer as a promising anode material for lithium-ion batteries. Chem. Commun. **2016**, *52*, 2035.

[7] Nie, P. *et al.* Prussian blue analogues: a new class of anode materials for lithium ion batteries. J. Mater. Chem. A **2014**, *2*, 5852.

[8] Li, C. *et al.* High anodic performance of co 1, 3, 5-benzenetricarboxylate coordination polymers for li-ion battery. ACS Appl. Mater. Interfaces **2016**, *8*, 15352.

[9] Wei, T. *et al.* POM-based metal-organic framework/reduced graphene oxide nanocomposites with hybrid behavior of battery-supercapacitor for superior lithium storage. Nano Energy **2017**, *34*, 205.

[10] Zhao, L. *et al.* Interdiffusion Reaction-Assisted Hybridization of Two-Dimensional Metal-Organic Frameworks and Ti3C2Tx Nanosheets for Electrocatalytic Oxygen Evolution. ACS Nano **2017**, *11*, 5800.

[11] Liu, S. H. *et al.* Meta-Organic-Framework-Derived Hybrid Carbon Nanocages as a Bifunctional Electrocatalyst for Oxygen Reduction and Evolution. Adv. Mater. **2017**, *29*, 1700874.

[12] Gao, J. K. *et al.* Bimetallic Hofmann-Type Metal-Organic Framework Nanoparticles for Efficient Electrocatalysis of Oxygen Evolution Reaction. ACS Appl. Energy Mater. **2018**, *1*. 5140.

[13] Wang, X. L. *et al.* Exploring the Performance Improvement of the Oxygen Evolution Reaction in a Stable Bimetal-Organic Framework System. Angew. Chem. Int. Ed. **2018**, *57*, 9660.

[14] Zhao S. Y. *et al.* Ultrathin metal-organic framework nanosheets for electrocatalytic oxygen evolution. Nat. Energy, **2016**, *1*, 1.

[15] Ma, T. Y., Dai, S., Jaroniec, M., Qiao, S. Z. Metal-Organic Framework Derived Hybrid Co3O4-Carbon Porous Nanowire Arrays as Reversible Oxygen Evolution Electrodes. J. Am. Chem. Soc. **2014**, *136*, 13925.

[16] Xia, Y. B. *et al.* A Metal-Organic Framework-Derived Bifunctional Oxygen Electrocatalyst. Nat. Energy **2016**, *1*. 1.

[17] Wurster, B., Grumelli, D., Hötger, D., Gutzler, R., Kern, K. Driving the Oxygen Evolution Reaction by Nonlinear Cooperativity in Bimetallic Coordination Catalysts. J. Am. Chem. Soc. **2016**, *138*, 3623.

[18] Wang, S., Hou, Y., Lin, S., Wang, X. Water Oxidation Electrocatalysis by a Zeolitic Imidazolate Framework. Nanoscale **2014**, *6*, 9930.

[19] Lu, X. F. *et al.* An Alkaline-Stable, Metal Hydroxide Mimicking Metal-Organic Framework for Efficient Electrocatalytic Oxygen Evolution. J. Am. Chem. Soc. **2016**, *138*, 8336.
